# Expression of RUNX1::RUNX1T1 deregulates the proteome, induces C/EBPβ suppression, and blocks myeloid differentiation

**DOI:** 10.1016/j.bneo.2026.100259

**Published:** 2026-06-18

**Authors:** Aleksandra Azevedo, Adam Leckenby, Rachael Nicholson, Ana Catarina Menezes, Sara Davies, Amanda F. Gilkes, Sarah Baker, Raymond Toghill, Ellie McCracken, Andrew Pierce, Bethany Geary, Anthony D. Whetton, Richard L. Darley, Alex Tonks

**Affiliations:** 1 Division of Cancer and Genetics, Department of Haematology, School of Medicine, Cardiff University, Cardiff, United Kingdom; 2 Cardiff Experimental and Cancer Medicine Centre, School of Medicine, Cardiff University, Cardiff, United Kingdom; 3 Department of Haematology, University Hospital of Wales, Cardiff, United Kingdom; 4 Stem Cell and Leukaemia Proteomics Laboratory, School of Medical Sciences, Faculty of Biology, Medicine and Health, The University of Manchester, Manchester, United Kingdom; 5 School of Medical and Health Sciences, Bangor University, Bangor, United Kingdom; 6 Proteomics Platform, UK Dementia Research Institute, University of Dundee, Dundee, United Kingdom; 7 Medical Research Council Protein Phosphorylation and Ubiquitylation Unit, University of Dundee, Dundee, United Kingdom; 8 Veterinary Health Innovation Engine, School of Veterinary Medicine, University of Surrey, Guildford, United Kingdom

## Abstract

Expression of RUNX1::RUNX1T1 (also known as RUNX1::ETO) is frequently observed in acute myeloid leukemia (AML) and has been shown to block myeloid development. Although several studies have shown RUNX1::RUNX1T1 transcriptional deregulation, the proteome in human hematopoietic stem and progenitor cells (HSPCs) is poorly characterized. Using mass spectrometry, we show that expression of RUNX1::RUNX1T1 in human HSPCs was linked to differential expression of 257 proteins, including CCAAT/enhancer-binding protein beta (C/EBPβ) downregulation. Consistent with this observation, although *CEBPB* mRNA is generally overexpressed in AML, patients with t(8;21) have comparatively low *CEBPB* expression. We show in human HSPCs that ectopic expression of C/EBPβ can promote proliferation of myeloid cells. Conversely, short hairpin RNA–mediated knockdown of C/EBPβ inhibited the growth of normal human myeloid cells; however, it promoted the growth of cells expressing RUNX1::RUNX1T1. C/EBPβ also influenced differentiation, with ectopic expression promoting monocyte development, whereas reduced expression favored granulocyte differentiation, which in turn suggests that C/EBPβ expression may act as a lineage discriminator for these myeloid cell types. In summary, our data reveal how RUNX1::RUNX1T1 drives deregulation of the HSPC proteome and suggest a mechanism by which RUNX1::RUNX1T1 expression contributes to leukemia development, including C/EBPβ, which itself affects normal myeloid development.

## Key Points

• RUNX1::RUNX1T1 alters the proteome of human HSPCs; mass spectrometry shows 257 proteins affected, including CEBPβ downregulation.
• CEBPβ expression is required for myeloid lineage choice and development.

## Visual Abstract

**Figure:**
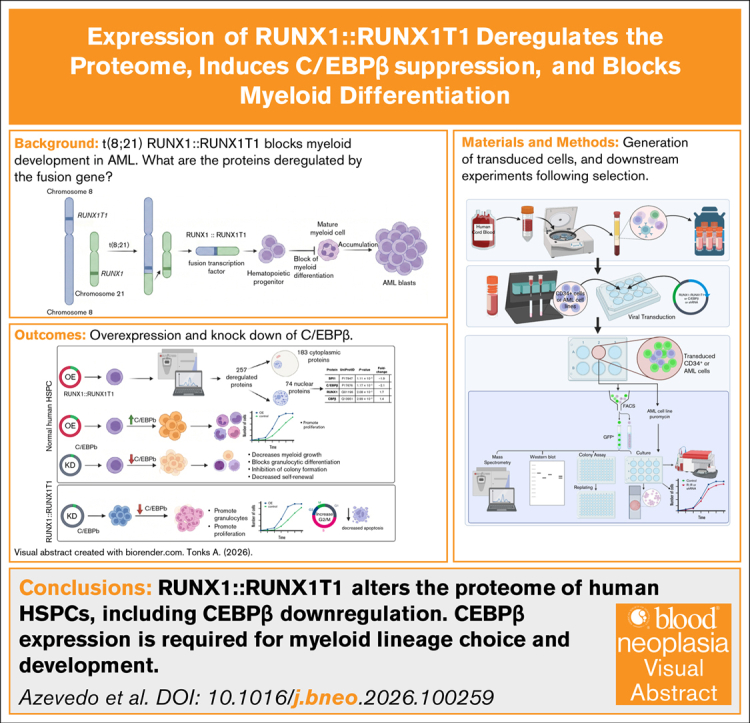

## Introduction

Acute myeloid leukemia (AML) is a hematologic malignancy characterized by several molecular abnormalities that ultimately lead to a block in hematopoietic differentiation.^1^ The reciprocal translocation t(8;21)(q22;q22) is one of the most frequent chromosomal abnormalities observed in AML and is responsible for expression of the fusion protein RUNX1::RUNX1T1 (also known as RUNX1::ETO).^2^ Patients with t(8;21) AML are often associated with better prognosis compared to patients with other subtypes of AML.^3^ However, a significant proportion of these patients eventually relapse.^4^^,^^5^ Therefore, new, less toxic, and more effective therapies are urgently required.

Previous studies using normal human primary hematopoietic stem and progenitor cells (CD34^+^ HSPCs) have shown that RUNX1::RUNX1T1 expression inhibits normal granulocytic development while promoting the development of immature blood cells with increased self-renewal potential.^6^, ^7^, ^8^ Other studies have shown that this fusion protein disturbs several transcriptional processes responsible for regulating normal hematopoiesis, including influencing myeloid transcription factor (TF) expression, such as SPI1 (also known as PU.1) and CCAAT/enhancer-binding protein alpha (C/EBPα).^9^, ^10^, ^11^ Many of these studies have used transcriptomic approaches to measure mRNA abundance as a strategy for target identification^11^^,^^12^; however, mRNA-level changes often do not correlate with protein expression changes.^13^^,^^14^ Further, the proteomic studies that have been performed in t(8;21) AML have used cell lines and/or murine models that may not be representative of human primary hematopoietic cells.^15^^,^^16^

Advances in mass spectrometry (MS) acquisition, including the development of sequential window acquisition of all theoretical mass spectra (SWATH-MS), have allowed investigators to identify and quantify proteins at a proteomic scale.^17^ The aim of this study was to determine quantitative changes in the proteomic profile of normal human CD34^+^-derived HSPCs expressing RUNX1::RUNX1T1. We quantified 4635 proteins, of which 257 were significantly differentially expressed in normal human HSPCs expressing RUNX1::RUNX1T1 compared with control. This approach identified not only known abnormally expressed TFs in primary human hematopoietic cells, such as SPI1 and CBFβ, but also novel abnormalities, including CCAAT/enhancer-binding protein beta (C/EBPβ), a member of the C/EBP family of TFs. Here, we investigated C/EBPβ expression in normal human primary hematopoietic cells and AML and determined its role in myelopoiesis. In summary, our data reveal how RUNX1::RUNX1T1 drives deregulation of the HSPC proteome and suggest a mechanism by which RUNX1::RUNX1T1 expression contributes to leukemia development, including C/EBPβ, which itself affects normal myeloid development.

## Methods

### Cell culture

AML cell lines were purchased and cultured according to recommended conditions (see the supplemental Methods). Fresh cord blood was also purchased from the National Health Service Bone Marrow and Transplant Unit (London, United Kingdom). Normal human CD34^+^ HSPCs were isolated using MiniMACS (Miltenyi Biotec, United Kingdom) as previously described and cultured in Iscove modified Dulbecco medium (Fisher Scientific, Loughborough, United Kingdom) supplemented with a combination of interleukin-3, stem cell factor, granulocyte colony-stimulating factor, and granulocyte-macrophage colony-stimulating factor (BioLegend, London, United Kingdom) at 5 ng/mL for up to 17 days to support myeloid development.^8^

### Generation of transduced HSPC and AML cell lines

A PINCO retroviral plasmid harboring RUNX1::RUNX1T1 complementary DNA and green fluorescent protein (GFP) was used to express RUNX1::RUNX1T1 in normal human CD34^+^ HSPCs; PINCO-GFP lacking RUNX1::RUNX1T1 was used as a control, as previously described.^6^ These unfractionated CD34^+^ HSPCs coexpress CD38 in >90% of cells isolated, meaning that they have lost lymphoid potential.^18^ C/EBPβ overexpression lentiviral vectors and short hairpin RNA (shRNA) lentiviral vectors with GFP and puromycin selectable markers (and relevant controls) were designed and purchased from VectorBuilder (Edinburgh, United Kingdom) (supplemental Methods). Normal human CD34^+^ HSPCs and cell lines were transduced on days 1 and 2 with virus as previously described.^6^^,^^8^^,^^11^ After transduction of HSPCs (day 3 of culture, when cells are >95% CD34^+^ and CD38^-^),^18^ cells were maintained in bulk-liquid culture for growth and differentiation assays using flow cytometry, or proteins were extracted for MS (see the following text). Where indicated, HSPCs were sorted by fluorescence-activated cell sorting for GFP positivity using FACSAria II (BD Biosciences, United Kingdom). Transduced AML cell lines were selected with puromycin (Sigma) at 1 μg/mL until control lines were all dead.

### Hematopoietic differentiation and morphological analysis

To assess myeloid cell differentiation, immunostaining coupled with flow cytometry was performed over time using a panel of specific cell surface markers (supplemental Methods).^19^ Cells were analyzed using the gating strategy outlined in supplemental Figure 1. Morphology was assessed on day 17 of culture (supplemental Methods).

### Colony-forming assay

Myeloid colony-forming efficiency assays were performed using a limiting dilution assay (0.3 cells per well) on GFP^+^ CD34^+^ HSPCs sorted by fluorescence-activated cell sorting in 96-U plates. Individual cells were cultured in Iscove modified Dulbecco medium as described earlier and incubated for 7 days at 37°C with 5% carbon dioxide (plating 1). Myeloid colonies (>50 cells) and clusters (>5 cells) were counted by light microscopy. To assess replating efficiency, identified colonies were harvested, counted, and replated (plating 2: 1 replating cycle only) at a density of 1 cell per well and cultured for an additional 7 days before scoring as earlier.

### Protein analyses

Protein extraction and western blotting were performed with the following primary antibodies: anti-AML1 (catalog no. 4334, Cell Signaling Technology, London, United Kingdom), anti-C/EBPβ (catalog no. 90081, Cell Signaling Technology), glyceraldehyde-3-phosphate dehydrogenase (GA1R, Fisher Scientific), and histone H3 (AE-4, Santa Cruz Biotechnology, Heidelberg, Germany), as previously described^13^^,^^19^ and in the supplemental Methods.

Detailed MS proteomic methods and data analysis are described in the supplemental Methods.

### Statistical analysis

Statistical and data analyses are detailed in the supplemental Methods. A paired-samples *t* test, assuming equal variances, a 1-way analysis of variance with Bonferroni multiple test correction, or a Mann-Whitney test was used unless otherwise stated. All statistical analyses were conducted using GraphPad Prism version 8 (GraphPad Software, California, United States).

Human neonatal cord blood was obtained after informed consent at the University Hospital of Wales, United Kingdom, with approval from the South-East Wales research ethics committee (06/WSE03/6) in accordance with the Declaration of Helsinki.

## Results

### RUNX1::RUNX1T1 expression in human CD34^+^ HSPC leads to C/EBPβ downregulation

**Figure 1.**
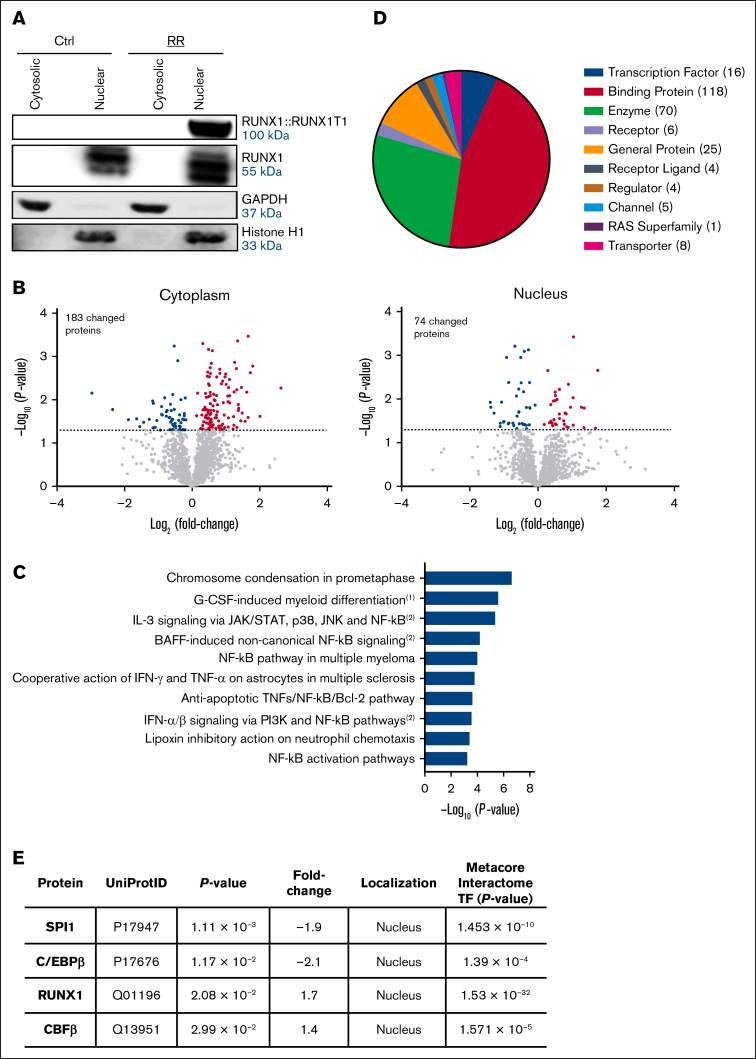
**Expression of RUNX1::RUNX1T1 in human CD34^+^ HSPCs alters the proteome, including TF expression.** (A) Western blot showing protein expression of RUNX1 and RUNX1::RUNX1T1 in CD34^+^ HSPCs transduced with a viral vector encoding RUNX1::RUNX1T1 or control (GFP alone) on day 3 of culture. Anti-RUNX1 antibody was used to detect the N terminus (containing RUNX1) of the RUNX1::RUNX1T1 fusion protein. Glyceraldehyde-3-phosphate dehydrogenase (GAPDH) and histone H1 were used as loading controls and to estimate subcellular fractionation efficiency for cytosolic and nuclear samples, respectively. Full-length blots are shown in supplemental Figure 4. (B) Volcano plots showing proteins differentially expressed in the cytoplasm (left panel) and nucleus (right panel) of RUNX1::RUNX1T1-expressing HSPCs compared to control (extracts isolated on day 3 of culture after infection). The y-axis represents statistical significance (-log_10_*P* value), whereas the x-axis indicates fold change (log_2_ fold change) of protein expression (n = 3). Statistical significance cutoff (*P* < .05; analysis of variance [ANOVA]) is highlighted with a horizontal dotted line. Blue and red color coding indicate significantly repressed and overexpressed proteins, respectively, in RUNX1::RUNX1T1 HSPCs compared to control. Gray indicates nonsignificant proteins. (C) Enrichment analysis of the 257 proteins (cytosolic and nuclear) identified as significantly dysregulated in HSPCs expressing RUNX1::RUNX1T1 compared with control, using Metacore. Top 10 significantly dysregulated pathway maps, ranked by -log (*P* value). An FDR of 0.05 was applied to the analysis. (D) Pie chart indicating the classification of the differentially expressed proteins using Metacore. (E) To identify significantly overconnected proteins within the data set, the Metacore “Interactome Transcription Factors” algorithm was used. Fold change represents protein upregulation or downregulation (negative values) in RUNX1::RUNX1T1 HSPCs compared with control. “Interactome TF” *P* values were calculated by the software. FDR, false discovery rate; RR, RUNX1::RUNX1T1.

To compare the abundance of cytoplasmic and nuclear proteins in RUNX1::RUNX1T1-expressing CD34^+^ HSPCs with corresponding control cells, nuclear and cytosolic proteins were extracted on day 3 of culture (when differentiation had not yet been affected by RUNX1::RUNX1T1) and analyzed by SWATH-MS (see supplemental Figure 2 for experimental design). Frequency of retroviral infection is shown in supplementary Figure 3. Western blot analysis of RUNX1::RUNX1T1 protein expression (using anti-RUNX1) revealed expression in the nucleus of RUNX1::RUNX1T1-transduced HSPCs, whereas no detectable fusion protein expression was observed in control cells (Figure 1A; supplemental Figure 4). Total protein detection after electrophoresis did not reveal protein sample degradation (supplemental Figure 5A). After SWATH-MS data acquisition and processing, as expected, glyceraldehyde-3-phosphate dehydrogenase protein was found almost exclusively in the cytoplasm of both control and RUNX1::RUNX1T1-expressing cells, whereas histone H1 was solely detected in the nuclear compartment (supplemental Figure 5B), further supporting the integrity of subcellular fractionation for SWATH-MS analysis. Taken together, these data reveal that we were able to generate high-quality subcellular protein extracts of RUNX1::RUNX1T1-expressing HSPCs and control cells.

Ectopic expression of RUNX1::RUNX1T1 led to modulation of 257 proteins, of which 183 proteins were changed in the cytoplasmic fractions and 74 were changed in the nucleus of RUNX1::RUNX1T1-expressing cells (Figure 1B; supplemental Table 1). Pathway enrichment analysis of these 257 proteins identified several dysregulated pathways, including NF-κB signaling, interleukin-3 signaling, and myeloid differentiation (Figure 1C), consistent with previous reports.^6^^,^^7^^,^^20^ To clarify the classes of proteins dysregulated by RUNX1::RUNX1T1, proteins were analyzed for their attributed function in Metacore (Figure 1D). Binding proteins and enzymes represented more than half of the total number of proteins significantly altered by expression of RUNX1::RUNX1T1. We identified 16 statistically significantly dysregulated TF proteins arising from RUNX1::RUNX1T1 expression (supplemental Figure 6). Of these, 11 TFs were upregulated, whereas 5 were downregulated in RUNX1::RUNX1T1 cells compared with controls. As expected, most of the dysregulated TFs were localized in the nucleus; however, 4 were found to have significantly dysregulated expression only in the cytoplasm (supplemental Figure 6).

To identify TF connectivity with other proteins from the data set, this study used the Metacore algorithm “Interactome Transcription Factors” on the significantly perturbed proteins (257 proteins). Four overconnected nuclear-expressed TFs were identified (Figure 1E; supplemental Figure 6). In agreement with previous studies, CBFβ and RUNX1 were found to be 2 of the TFs with the highest number of interactions with other proteins from the data set.^21^ Further, we identified another known TF to be dysregulated: SPI1, a master regulator of myeloid cell development.^10^ Importantly, we identified a 2.1-fold reduction in nuclear expression level of C/EBPβ (*CEBPB*) in RUNX1::RUNX1T1-expressing HSPCs compared with control (Figure 1E; supplemental Figure 7A). Using a previous transcriptome data set, *CEBPB* mRNA was also found to be significantly repressed in RUNX1::RUNX1T1-expressing cells^11^ (supplemental Figure 7B, microarray data set E-MEXP-583). This protein is a member of the C/EBP family, which has been known to play important roles in hematopoietic proliferation and differentiation,^22^ including emergency granulopoiesis,^23^ and the suppression of myeloid leukemogenesis.^24^^,^^25^ To date, no human studies have been performed to determine the role of C/EBPβ in normal human hematopoiesis or in patients with AML with t(8;21).

### *CEBPB* mRNA is expressed at lower levels in t(8;21) AML compared to other AML subtypes

**Figure 2.**
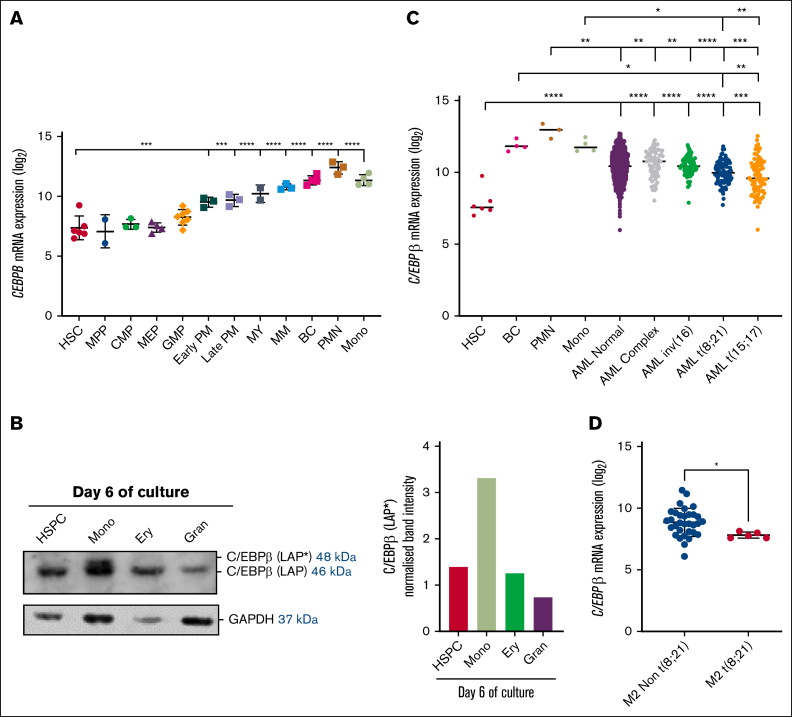
**C/EBPβ expression in normal human myeloid subpopulations and AML.** (A) *CEBPB* mRNA expression in distinct normal human hematopoietic cellular subsets, based on specific cell surface marker expression. Expression levels from GSE24759.^29^ Data indicate mean ± 1 standard deviation (SD) (n ≥ 2). Significant differences between each cell subset and HSCs were analyzed by 1-way ANOVA with Bonferroni multiple-comparison correction; ∗*P* < .05; ∗∗*P* < .01; ∗∗∗*P* < .001; ∗∗∗∗*P* < .0001. Probe set 202501_at was used to quantitate levels of *CEBPB* mRNA. (B) Western blot analysis showing C/EBPβ expression in human HSPCs (day 3) and in monocytic (CD14^high^), erythroid (CD14^low^CD36^high^), and granulocytic (CD14^low^CD36^low^) progenitor cells cultured toward day 6 of culture; lineage fractions were isolated from human cord blood as described in Tonks et al.^11^ GAPDH was used as a loading control. C/EBPβ LAP∗ or FL (see “Discussion”). Full immunoblot is shown in supplemental Figure 8. C/EBPβ expression was quantified using ImageJ and normalized to loading control (GAPDH) (n = 1). (C) *CEBPB* mRNA expression in normal human hematopoietic subsets vs AML subtypes. Normal human hematopoiesis data were derived from GSE422519; human AML cells were derived from GSE13159, GSE15434, GSE61804, GSE14468, and The Cancer Genome Atlas (TCGA) (data analyzed using BloodSpot BloodPool algorithm^26^). Data indicate mean ± 1 SD (n ≥ 3). Significant differences between each cell subset and AML subtypes were analyzed by 1-way ANOVA with Bonferroni multiple-comparison correction; ∗*P* < .05; ∗∗*P* < .01; ∗∗∗*P* < .001; ∗∗∗∗*P* < .0001. Probe set 202501_at was used to quantitate the level of *CEBPB* mRNA. (D) *CEBPB* mRNA expression in FAB-M2 AML, categorized into t(8;21) and non-t(8;21). Data were obtained from TCGA.^30^ Data indicate mean ± 1 SD (n ≥ 5). Significant differences were analyzed by unpaired *t* test; ∗*P* < .05; ∗∗*P* < .01; ∗∗∗*P* < .001; ∗∗∗∗*P* < .0001. BC, band cell; Ery, erythrocytes; FAB, French-American-British; FL, full-length; Gran, granulocytes; MEP, megakaryocyte-erythroid progenitor; MM, metamyelocyte; MPP, multipotent progenitor; MY, myelocyte; PM, promyelocyte; PNM, polymorphonuclear cell.

To determine the expression of *CEBPB* mRNA in human hematopoietic cells, publicly available microarray data were analyzed using the online repository BloodSpot.^26^ Figure 2A shows that expression of *CEBPB* mRNA is lower in less differentiated cells, including hematopoietic stem cells (HSCs), multipotent progenitors, and common myeloid progenitors, than in differentiated granulocytes and monocytes. These observations suggest transcriptional regulation of *CEBPB* during hematopoietic development. To validate mRNA expression data, C/EBPβ protein expression in each lineage was assessed in total protein lysates from monocytic-, granulocytic-, and erythroid-committed progenitor cells derived from culture of CD34^+^ HSPCs under specific culture conditions previously described.^11^ *CEBPB* is an intronless gene consisting of 1 exon and located on chromosome 20q13.13, coding to produce a single mRNA molecule.^27^ However, because of the presence of 3 initiation codons at different sites,^27^^,^^28^ it can generate 3 protein isoforms: liver-enriched activating protein∗ (LAP∗ or full-length), liver-enriched activating protein (LAP), and liver-enriched inhibitory protein (LIP). We found LAP expression to be dominant in the monocytic lineage, with similar levels in other lineages tested (Figure 2B; supplemental Figure 8). Taken together, the data suggest that C/EBPβ is differentially expressed in the different myeloid progenitor-committed populations, which indicates a role for this protein in myeloid cell development.

To determine the expression of *CEBPB* mRNA in different subtypes of AML, we analyzed publicly available transcriptomic data sets and compared them with normal undifferentiated HSCs and mature granulocytes/monocytes. Figure 2C shows that *CEBPB* mRNA expression is significantly increased in all subtypes of AML studied, compared with normal HSCs. However, mRNA expression was significantly lower compared with normal terminally differentiated myeloid cells, suggesting that reduced *CEBPB* expression might contribute to a block in normal hematopoietic differentiation. Interestingly, within the French-American-British M2 AML subtype, *CEBPB* expression was found to be significantly lower in patients with t(8;21) compared to patients with non-t(8;21) French-American-British M2 AML (Figure 2D), suggesting a role for this gene in the development of AML t(8;21).

We next analyzed The Cancer Genome Atlas (TCGA) 2013 RNASeq data set^30^ to determine overall survival and disease-free survival of patients with non–acute promyelocytic leukemia (APL) AML who received intensive therapy and had high and low *CEBPB* expression. Patients were stratified according to *CEBPB* mRNA expression using the upper and lower quartiles (high and low *CEBPB* mRNA expression, respectively) (supplemental Figure 9A). There were no significant differences in the median survival of patients with AML with high *CEBPB* expression compared to low *CEBPB* mRNA expression (supplemental Figure 9B). However, high *CEBPB* expression was associated with a significant decrease in disease-free survival of 9.25 months, compared to 39 months for patients with low *CEBPB* mRNA expression (supplemental Figure 9C). Correspondingly, an increased risk of relapse was also observed (hazard ratio, 2.4; 95% confidence interval, 1.2-4.8). Interestingly, t(8;21) was solely linked to low *CEBPB* expression (supplemental Figure 10A), which was also significantly associated with mutations in both the *RUNX1* and *RUNX1T1* genes (supplemental Figure 10B). These observations further strengthen the hypothesis that C/EBPβ downregulation plays a role in the development of AML t(8;21).

### KD of C/EBPβ decreases normal human myeloid growth and blocks terminal granulocytic development

We next determined the requirement of C/EBPβ for normal human hematopoietic growth and development using lentiviral shRNA knockdown (KD). Having validated the expression of C/EBPβ LAP∗ and LAP in normal human HSPCs, we screened 3 KD vectors for their efficiency in reducing expression of C/EBPβ (supplemental Figure 11). shC/EBPβ 2 and 3 showed a 40% to 50% reduction of LAP∗ protein expression and a 45% reduction in LAP compared to shRNA control (supplemental Figure 11). In contrast, shC/EBPβ 1 had no detectable effect on endogenous levels of the C/EBPβ protein and was consequently excluded from subsequent analysis.

**Figure 3.**
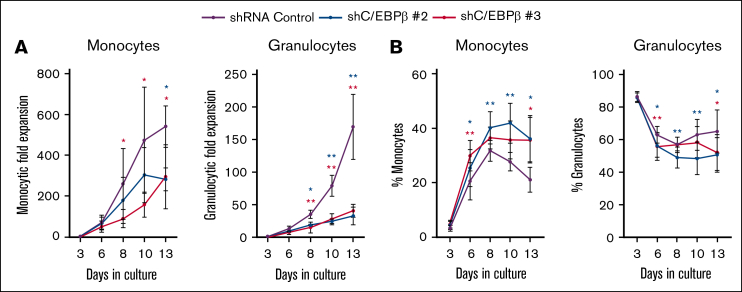
**KD of C/EBPβ inhibits granulocytic and monocytic growth and disrupts the balance between monocytic and granulocytic populations during normal myeloid development.** (A) Cumulative fold expansion of control and shC/EBPβ myeloid cultures in terms of monocytic-committed cells (CD13^high^CD36^high^; left panel) and granulocytic-committed cells (CD13^low/high^CD36^low^; right panel) cultured over 13 days in medium containing interleukin-3 (IL-3), stem cell factor (SCF), granulocyte colony-stimulating factor (G-CSF), and granulocyte-macrophage colony-stimulating factor (GM-CSF). (B) Summary data of the percentage of monocytic-committed cells (CD13^high^CD36^high^; left panel) and granulocytic-committed cells (CD13^low/high^CD36^low^; right panel) in control and shC/EBPβ cultures. Data indicate mean ± 1 SD (n = 4). Significant differences between control and shC/EBPβ cultures were analyzed by 1-way ANOVA with Bonferroni multiple-comparison correction; ∗*P* < .05; ∗∗*P* < .01.

**Figure 4.**
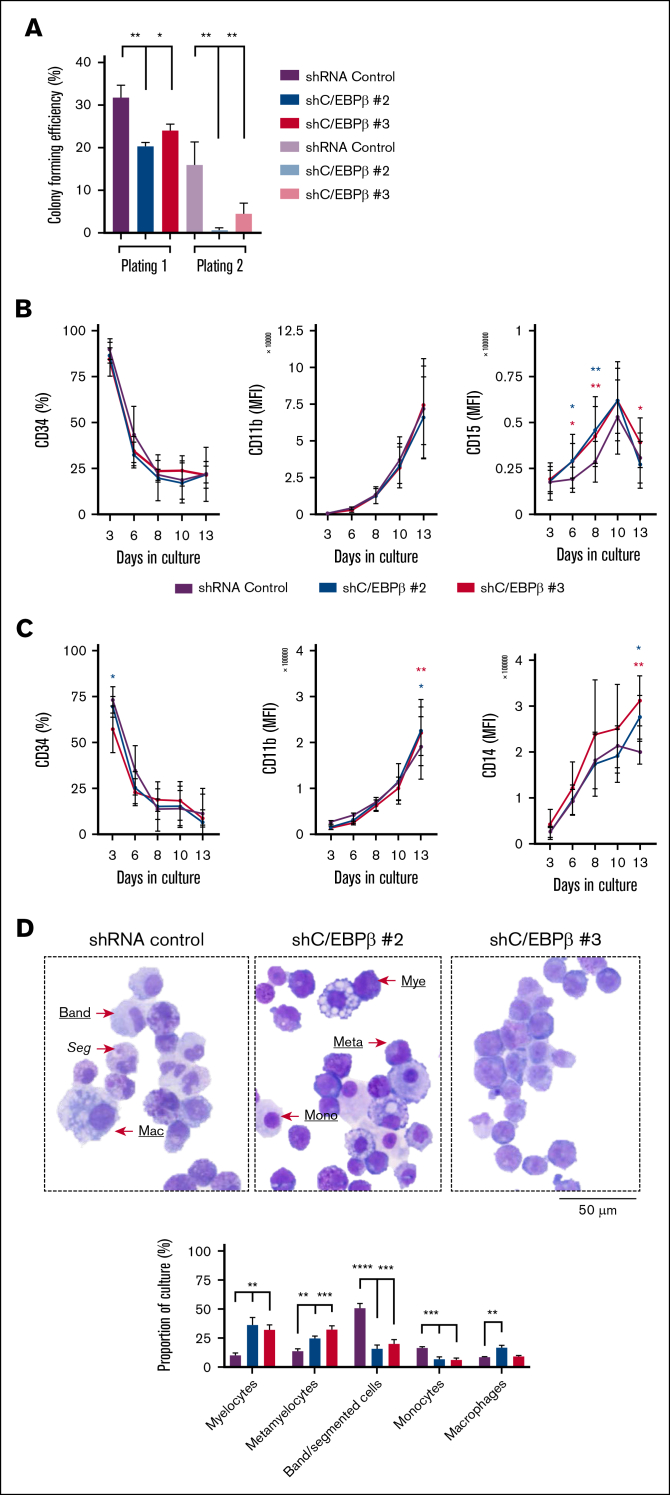
**KD of C/EBPβ promotes the expression of myeloid cell surface markers and alters cell morphology during normal human myeloid differentiation.** (A) Summary data of myeloid colony-forming efficiency for control and shC/EBPβ cultures after 7 days of growth in liquid culture containing IL-3, SCF, G-CSF, and GM-CSF (plating 1, P1). Single transduced cultures were sorted for GFP positivity on day 3 by fluorescence-activated cell sorting (FACS). Self-renewal potential was assessed by a single replating round of control and shC/EBPβ cultures in the same conditions as previously (plating 2, P2). (B) Summary data of granulocytic development (GFP^+^, CD13^low/high^CD36^low^) of control and shC/EBPβ cultures in terms of CD34 percentage (left panel), CD11b expression (middle panel), and CD15 expression (right panel), grown over 13 days in culture medium. (C) Summary data of monocytic development (GFP^+^, CD13^high^CD36^high^) of control and shC/EBPβ cultures in terms of CD34 percentage (left panel), CD11b expression (middle panel), and CD14 expression (right panel), grown over 13 days in culture medium. Data indicate mean ± 1 SD (n = 4). (D) Control and shC/EBPβ cultures were analyzed on day 17 of differentiation using May-Grünwald-Giemsa staining (upper panels); differential counts of all cultures with morphology were categorized into granulocytic- and monocytic-committed cells (lower panel). Granulocytic cells were further divided into myelocytes, metamyelocytes, and band/segmented cells. Data indicate mean ± 1 SD (n = 3). Significant differences between control and shC/EBPβ cultures were analyzed by 1-way ANOVA with Bonferroni multiple-comparison correction; ∗*P* < .05; ∗∗*P* < .01; ∗∗∗*P* < .001; ∗∗∗∗*P* < .0001.

We next analyzed the effect of C/EBPβ KD on the proliferation and differentiation of the different myeloid lineages. For this, CD13 and CD36 cell surface markers were used to discriminate among monocytic, granulocytic, and erythroid populations (supplemental Figure 1). As shown in Figure 3A, the cumulative growth of monocytic and granulocytic cells was significantly impaired (by twofold and fivefold, respectively, at day 13). This had the effect of increasing the relative proportion of monocytes in the culture at the expense of granulocytic cells (Figure 3B). We also assessed colony replating capacity; under clonal conditions, both C/EBPβ KD cultures had significantly fewer myeloid colonies compared to control, showing a reduction of 25% to 36% (Figure 4A). Moreover, both KD cultures showed a significant decrease in replating capacity. Collectively, these observations suggest that KD of C/EBPβ affects the growth of myeloid cells. To analyze the consequences of C/EBPβ KD on monocytic and granulocytic differentiation, we assessed the expression of the cell surface markers CD34, CD11b, CD14, and CD15.^6^ Overall, there was little difference in the expression of CD34, CD11b, and CD14 (Figure 4B-C). However, shC/EBPβ KD showed significant upregulation of the granulocytic marker CD15 throughout myeloid development, suggesting promotion of granulocytic development (Figure 4B). Interestingly, shC/EBPβ KD cultures showed a significant decrease in the percentage of terminally differentiated granulocytic cells, suggesting a differentiation arrest (Figure 4D). Overall, these observations support a role for C/EBPβ in regulating granulocytic development. However, a potential role in monocyte development cannot be fully excluded based on level of KD, as well as the interplay with other members of the C/EBP family of TFs (see “Discussion”).

### C/EBPβ KD in t(8;21) AML cell line promotes cell growth and survival

**Figure 5.**
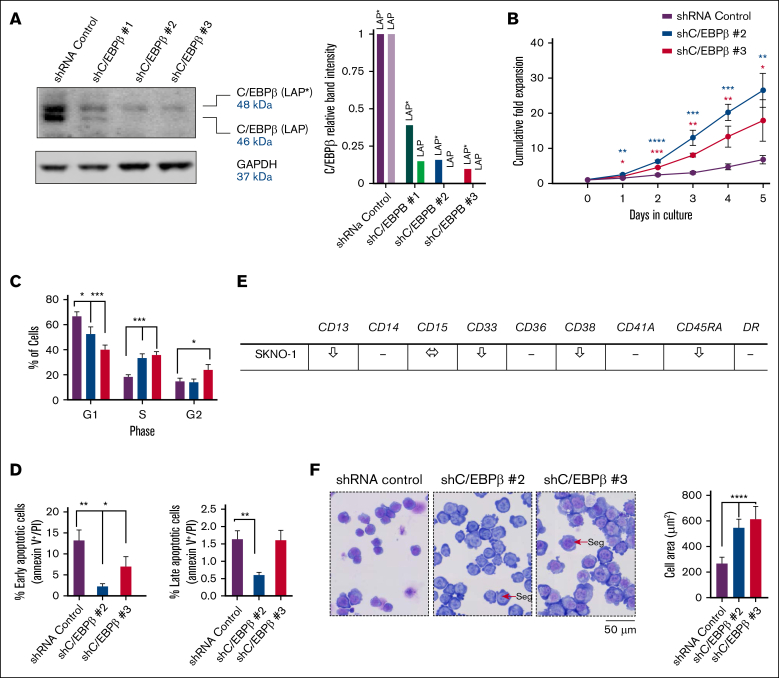
**KD of C/EBPβ in a t(8;21) AML cell line promotes a granulocytic phenotype.** (A) Western blot (left panel) analysis showing C/EBPβ protein expression in SKNO-1 cells transduced with control or shRNA to C/EBPβ. GAPDH was used as a loading control. Right panels show western blot quantification by densitometry analysis using ImageJ. Relative protein expression was determined by normalizing each sample first to the loading control (GAPDH), then to shRNA control (n = 1). (B) Summary data showing the growth of SKNO-1 cells with 2 shC/EBPβ constructs compared with control over 5 days of culture. (C) Summary data showing the effect of C/EBPβ KD on normal cell cycle in SKNO-1 cells. (D) Summary data showing the effect of C/EBPβ KD on normal apoptotic rate using annexin V staining in SKNO-1 cells. Bars indicate percentage of preapoptotic cells (annexin V^+^/7-AAD^-^) and late apoptotic cells (Annexin V^+^/7-AAD^+^). Data indicate mean ± 1 SD (n > 3). Significant differences between shC/EBPβ cultures and control were analyzed using 1-way ANOVA with Bonferroni multiple-comparison correction; ∗*P* < .05; ∗∗*P* < .01; ∗∗∗*P* < .001; ∗∗∗∗*P* < .0001. (E) Summary of phenotypic changes in SKNO-1 cells with C/EBPβ KD. Change in expression denotes comparison with control cell lines. Cell surface proteins not detected are not represented in this table. Variation in flow cytometric data sets is shown in supplemental Figure 12. Arrows indicate direction of change compared with control. Dash indicates no change. (F) Images showing morphology of SKNO-1 cells transduced with control or shC/EBPβ constructs. Bar chart (right panel) showing the area of SKNO-1 cells calculated using ImageJ. Arrows show segmented nuclei. Data indicate mean ± 1 SD (n = 3). Significant differences between shC/EBPβ cultures and control were analyzed using 1-way ANOVA with Bonferroni multiple-comparison correction; ∗∗*P* < .01; ∗∗∗∗*P* < .0001.

Changes in C/EBPβ expression have been frequently reported in the context of hematologic malignancy (see “Discussion”).^31^^,^^32^ To determine the consequences of modulating C/EBPβ expression in the context of t(8;21) AML, we examined the effect of overexpression and KD in SKNO-1 cells. We were unable to stably overexpress C/EBPβ in these cells despite clear transduction, suggesting that this is toxic, possibly by acting as a dominant transcriptional repressor of RUNX1::RUNX1T1.^33^ Correspondingly, our earlier observations would predict that reducing C/EBPβ expression may also affect growth and development; we therefore also investigated the impact of KD in this context. Constructs shC/EBPβ 2 and 3 decreased the detectable levels of C/EBPβ LAP∗ in SKNO-1 cells by ∼75% compared to control (Figure 5A). KD of C/EBPβ in SKNO-1 using shC/EBPβ 2 and 3 resulted in significant 4.0- and 2.6-fold increases, respectively, in cell growth (Figure 5B). Cell cycle analysis demonstrated a significant increase in the proportion of cells in the S/G2 phase of the cell cycle and a concurrent decrease in the proportion of cells in the G1 phase compared to control (Figure 5C). Further, a significant decrease in the percentage of cells in apoptosis was also observed compared to control (Figure 5D).

To determine whether KD of C/EBPβ had any effect on the differentiation status of transformed SKNO-1 cells, shC/EBPβ-transduced cells were analyzed using immunophenotypic assays. According to this analysis, SKNO-1 control cells are typically CD13^+^CD33^+^CD38^+^CD45RA^+^CD15^+^, resembling a granulocyte-monocyte progenitor phenotype (supplemental Figure 12). However, upon KD of C/EBPβ, these cells lost the expression of all myeloid markers except CD15 (summarized in Figure 5E) and showed signs of segmented nuclei, as well as being larger (Figure 5F). Together, these data suggest that KD of C/EBPβ in AML t(8;21) cell lines promotes a shift to a granulocytic development program consistent with its effect in normal primary cells; however, these data are limited and should be interpreted with caution.

### Overexpression of C/EBPβ influences normal myeloid development

**Figure 6.**
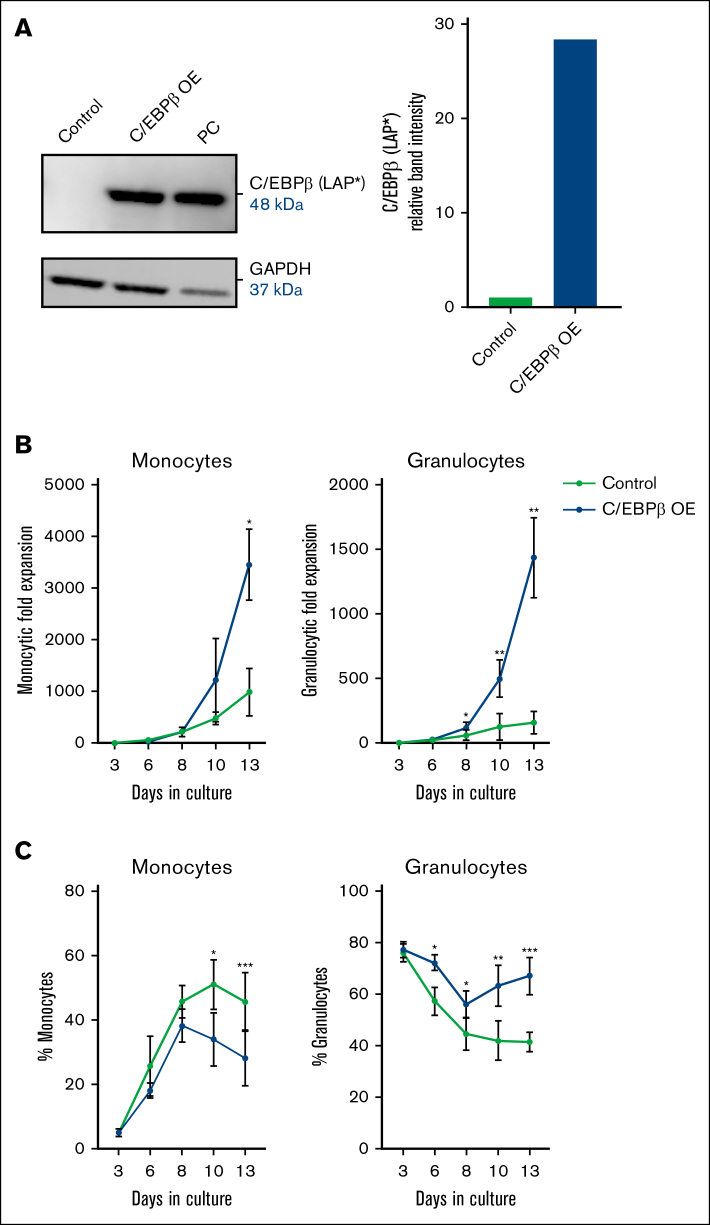
**Overexpression of C/EBPβ influences myeloid development.** (A) Left panel, western blot analysis showing C/EBPβ protein expression in FACS-sorted HSPC transduced with control or a C/EBPβ LAP∗ overexpression construct on day 15 of culture. HEK 293T cells transfected with a C/EBPβ-overexpression construct were used as a PC; GAPDH was used as a loading control. Full blot is depicted in supplemental Figure 13. Right panel, bar chart showing densitometry analysis of C/EBPβ expression in HSPCs transduced with a C/EBPβ LAP∗ overexpression construct. C/EBPβ expression was quantified using ImageJ and normalized to loading control (GAPDH) and to control (n = 1). (B) Summary data of the percentage of monocytic-committed cells (CD13^high^CD36^high^; left panel) and granulocytic-committed cells (CD13^low/high^CD36^low^; right panel) in control cultures and cells ectopically expressing C/EBPβ. (C) Cumulative fold expansion of control and C/EBPβ myeloid cultures in terms of monocytic-committed cells (CD13^high^CD36^high^; left panel) and granulocytic-committed cells (CD13^low/high^CD36^low^*;* right panel) cultured over 13 days in medium containing IL-3, SCF, G-CSF, and GM-CSF. Data indicate mean ± 1 SD (n = 4). Significant differences between control and C/EBPβ cultures were analyzed by paired t test; ∗*P* < .05; ∗∗*P* < .01; ∗∗∗*P* < .001. PC, positive control.

Although patients with t(8;21) exhibited lower levels of *CEBPB* mRNA than other AML subsets (excluding APL), expression of *CEBPB* mRNA was overall higher in AML than in normal HSCs. To understand the effect of C/EBPβ on normal hematopoiesis, we therefore analyzed the consequences of C/EBPβ overexpression in HSPCs using a C/EBPβ LAP∗ construct. Western blot analysis showed that C/EBPβ-transduced cells strongly overexpressed the C/EBPβ LAP∗ protein compared to control cells (Figure 6A; supplemental Figure 13) on day 15 of culture.

C/EBPβ-overexpressing monocytes displayed a 3.5-fold increase in growth compared to control cells (Figure 6B). This increase was more evident in the granulocytic population, in which cell expansion was increased by 9-fold in C/EBPβ-overexpressing cells compared with control cells (Figure 6B). Furthermore, overexpression of C/EBPβ significantly decreased the proportion of the monocytic population (Figure 6C), especially in the later stages of myeloid development, coupled with an increase in the percentage of granulocytic-committed cells throughout myeloid development (Figure 6C). These observations are reciprocal to the proliferative effect of C/EBPβ KD on monocyte and granulocyte growth (Figure 3).

**Figure 7.**
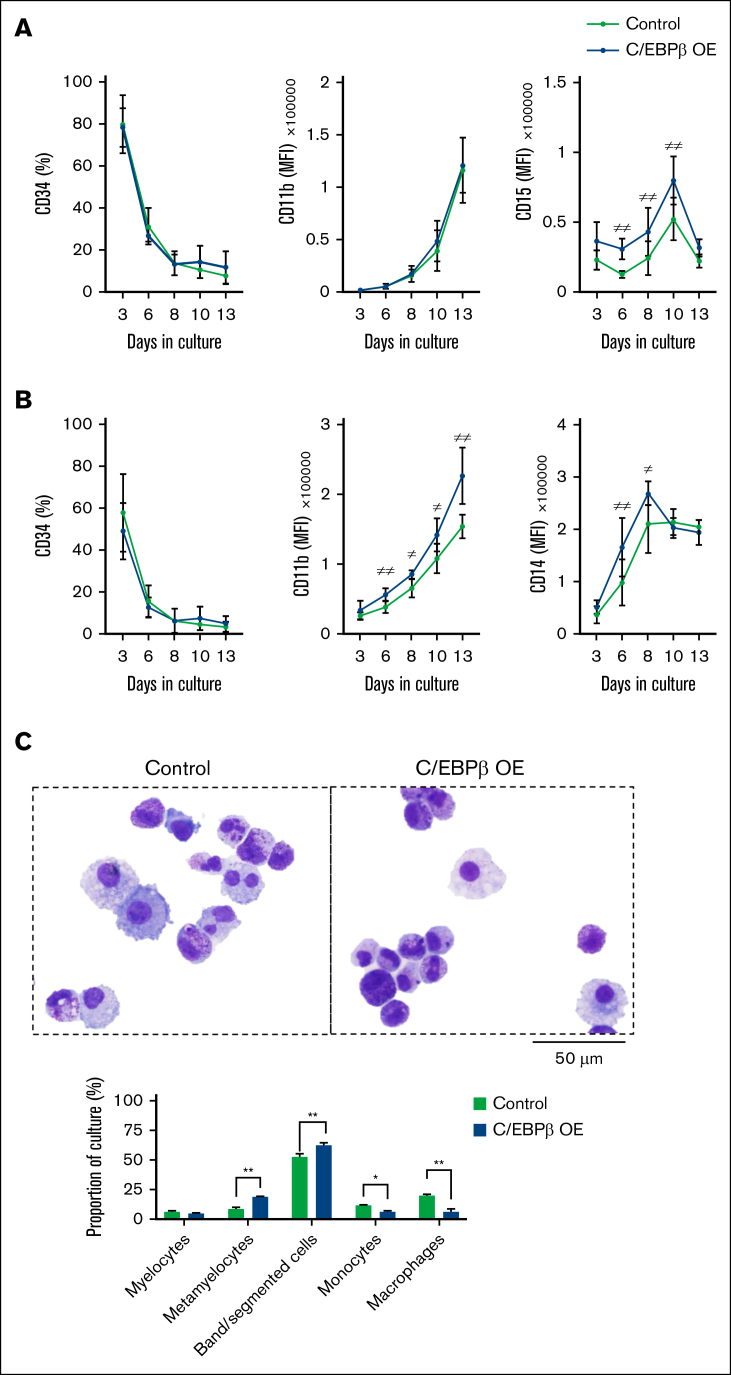
**Overexpression of C/EBPβ promotes the expression of myeloid cell surface markers and alters cell morphology during normal human myeloid differentiation.** (A) Summary data of granulocytic development (CD13^low/high^CD36^low^) of control and C/EBPβ overexpression cultures in terms of CD34 percentage (left panel), CD11b expression (middle panel), and CD15 expression (right panel), grown over 13 days in culture medium containing IL-3, SCF, G-SCF, and GM-CSF. (B) Summary data of monocytic development (CD13^high^CD36^high^) of control and C/EBPβ overexpression cultures in terms of CD34 percentage (left panel), CD11b expression (middle panel), and CD14 expression (right panel), grown over 13 days in culture medium. Data indicate mean ± 1 SD (n = 4). (C) Control and C/EBPβ overexpression cultures were analyzed on day 17 of differentiation using May-Grünwald-Giemsa staining (upper panel); differential counts of all cultures with morphology were categorized into granulocytic- and monocytic-committed cells (lower panel). Granulocytic cells were further divided into myelocytes, metamyelocytes, and band/segmented cells. Data indicate mean ± 1 SD (n = 3). Significant differences between control and C/EBPβ overexpression cultures were analyzed by paired *t* test; ∗*P* < .05; ∗∗*P* < .01.

Immunophenotypic analysis of monocyte progenitor cells revealed no difference in the percentage of cells expressing the pluripotent hematopoietic cell marker CD34 (Figure 7A). However, more rapid upregulation of CD11b and CD14 was observed (Figure 7A). This phenotype further agrees with mRNA and protein analyses, in which C/EBPβ expression increased with myeloid cell differentiation, specifically in monocytic-committed cells (Figure 3). In contrast, C/EBPβ overexpression did not significantly affect the expression of CD34 or CD11b in granulocytic progenitor cells (Figure 7B). Overexpression of C/EBPβ in this population did, however, lead to upregulation of the granulocytic marker CD15 until day 10 of culture, at which point 1.5-fold upregulation was observed compared with control cells (Figure 7B).

Morphological examination showed that overexpression of C/EBPβ promoted granulocytic development, with a significant increase in the percentage of cells showing mature granulocytic traits, such as segmented nuclei and cytoplasmic granules (Figure 7C). Moreover, the percentage of monocytes and macrophages in the C/EBPβ-overexpressing culture was significantly lower compared to the control population.

Altogether, these results suggest that ectopic expression of C/EBPβ LAP∗ in HSPC promotes proliferation of monocytic and granulocytic cells but does not inhibit terminal differentiation.

## Discussion

AML is a blood disorder characterized by the clonal expansion of immature HSPCs, with a blockade of terminal differentiation. Previously, RUNX1::RUNX1T1 has been shown to inhibit normal myeloid differentiation in mice.^2^ Furthermore, ectopic expression of RUNX1::RUNX1T1 in human HSPCs was shown to block granulocytic development and promote their self-renewal.^6^, ^7^, ^8^ However, despite the progress in understanding the biological implications of t(8;21), the mechanism through which this fusion protein leads to the development of AML is not yet fully understood. A better understanding of this mechanism would allow the identification of novel therapeutic targets for the treatment of this disease.

Although most studies have focused on RUNX1::RUNX1T1-mediated transcriptional changes,^11^^,^^34^^,^^35^ there is a paucity of information regarding analysis of the proteomic profile of cells in primary human cells. Transcriptional analysis does not account for mRNA transcriptional control, posttranscriptional or posttranslational changes, epigenetic regulators, or protein half-life, all of which can contribute to modulation of protein turnover and to changes in the balance between mRNA modulation and differential levels of protein expression. To address some of these shortcomings, we used SWATH-MS to quantify changes in the proteome of normal human cells upon expression of RUNX1::RUNX1T1. SWATH-MS allows the detection of thousands of proteins in a single run, maximizing the information obtained per sample while significantly decreasing the time used for each experiment.^36^ The main disadvantage of SWATH-MS is cost, which can constrain the use of MS-based techniques. Using this approach, we identified 257 differentially expressed proteins involved in several pathways previously described in AML, including dysregulation of normal myeloid differentiation. In addition, this approach was able to identify known abnormally expressed TFs, such as SPI1 and CBFβ. Importantly, we also identified novel abnormalities, including a reduction in nuclear expression level of C/EBPβ in RUNX1::RUNX1T1-expressing human primary HSPCs compared with control.

*CEBPB* is an intronless gene that is a member of the C/EBP family of TFs. This gene generates 3 C/EBPβ protein isoforms: LAP∗ (or full-length), LAP, and LIP.^37^ Although LAP∗ and LAP are considered transcriptional activators, LIP is regarded as a dominant inhibitor of transcriptionally active C/EBPs, including LAP∗ and LAP.^38^ Some authors have claimed that knocking out C/EBPβ in early mouse progenitor cells gives rise to a lower number of colonies and cells,^23^ whereas other studies have shown that knockout in more differentiated cells results in a proproliferative phenotype.^25^^,^^39^ Consistent with previous observations,^40^ we found that C/EBPβ was expressed across all human hematopoietic lineages, with higher expression observed in mature monocytes and lower levels of C/EBPβ protein in granulocyte progenitor cells. The assertion that C/EBPβ overexpression induces granulocytic differentiation has been previously reported under various experimental conditions^23^^,^^41^, ^42^, ^43^; however, our study extends these observations by identifying RUNX1::RUNX1T1-associated suppression of nuclear C/EBPβ protein as an early proteomic lesion in primary HSPCs.

A previous study using transformed U937 cells expressing RUNX1::RUNX1T1 revealed that expression of C/EBPβ is directly regulated by this fusion protein.^44^ It is tempting to speculate that because lower *CEBPB* mRNA expression is linked to better treatment response, this may contribute to why patients with t(8;21) have a favorable risk prognosis. C/EBPβ has also been implicated in the development of several myelomonocytic leukemias, including FMS-like tyrosine kinase 3 - Internal Tandem Duplication (FLT3-ITD)–mutated AML^31^ as well as in several other leukemic cell lines,^32^^,^^45^ where an increased concentration of LIP has been observed, leading to disease development. On the other hand, upregulation of LAP∗ and LAP has been associated with reduced cell proliferation and induction of differentiation in leukemic cell lines.^46^ Interestingly, we attempted to ectopically express C/EBPβ in RUNX1::RUNX1T1-expressing AML cells but were unable to overexpress the protein despite clear transduction. There are several biological and technical reasons that this was not possible. Given that we were able to ectopically express this protein in normal HSPCs, it is most likely a result of opposing the RUNX1::RUNX1T1 program, which is known to act as a dominant transcriptional repressor.^33^ However, we cannot rule out strong negative selection because of quiescence or cell death.

Although C/EBPβ is important for stress-induced granulopoiesis in mice,^47^^,^^48^ the functional implication of altered C/EBPβ expression in normal human cells remains unidentified. We determined the role of C/EBPβ in normal human myelopoiesis and context-dependent requirements for C/EBPβ in normal myelopoiesis and t(8;21) AML. Expression in HSPCs inhibited the growth of normal myeloid cells; however, in the context of RUNX1::RUNX1T1 expression in SKNO-1 cells, KD of C/EBPβ strongly promoted proliferation. Interestingly, in the BCR-ABL–expressing cell line 32D, forced expression of C/EBPβ led to a decrease in cell proliferation and promoted differentiation.^46^ We also found that C/EBPβ can influence differentiation, with evidence that ectopic expression promotes monocytic differentiation, whereas reduction in C/EBPβ expression promotes the expression of the granulocytic marker CD15, which in turn suggests that C/EBPβ may act as a lineage discriminator for these myeloid cell types. Interestingly, the complete abrogation of multiple markers indicated that the developmental status of these cells was also affected. It has been shown that C/EBPβ cooperates with MYB and the coactivator p300 to promote the expression of myeloid-associated genes.^49^ Previous studies have shown that, depending on cell context, the regulatory function of C/EBPβ is variable.^50^ For instance, knockout of C/EBPβ in mice resulted in a decrease in the number of B lymphocytes with reduced cell expansion properties.^51^ Developmentally, KD of C/EBPβ in HSPCs did not significantly alter the monocytic differentiation markers CD11b and CD14, showing that C/EBPβ was not required for the survival of mature monocytes, as suggested by Tamura et al in 2017.^52^ However, in that study, a mouse C/EBPβ^-/-^ model of peripheral blood monocyte survival was used, suggesting human vs murine differences. Alternatively, in the current study, the C/EBPβ KD level was ∼50%, and it is possible that these levels are sufficient to maintain survival.

Interestingly, a recent study reported that inhibition of C/EBPβ activity using 4,15-iso-atriplicolide derivatives promoted the expression of differentiation-associated cell surface markers and genes in AML cell lines.^53^ This contrasts with our observations, with the caveat that the specificity of this agent is not fully understood. It is important to note that functional redundancy with other members of the C/EBP family could explain why KD of C/EBPβ alone does not block granulocytic development, as was shown previously for cells expressing RUNX1::RUNX1T1.^6^ Other C/EBP isoforms might be acting compensatorily in the setting of C/EBPβ KD; in the hematopoietic system, C/EBPα, C/EBPβ, C/EBPδ, and C/EBPε are all expressed within the myeloid lineage.^54^ Overlapping expression of the C/EBP family members suggests that myeloid development is regulated by different combinations of C/EBP homo- and heterodimers. Several studies have shown that these TFs can act in a compensatory fashion in the absence of a certain isoform.^55^^,^^56^ This has been demonstrated in granulocytic development, in which C/EBPα and C/EBPβ collaborate with each other to ensure granulocyte production.^42^ It is also plausible that the effects observed would have been different if C/EBPβ had been knocked down in more differentiated cells, as opposed to HSPCs. Altogether, the phenotype recapitulates clinical features of t(8;21) and previous studies in which RUNX1::RUNX1T1 affects granulocytic development.^6^

We aimed to determine the effect of overexpressing C/EBPβ LAP∗ as a single abnormality on the myeloid development of human HSPCs. Our data suggest that ectopic expression of C/EBPβ LAP∗ in HSPCs promotes monocytic and granulocytic proliferation. However, this is not a result of inhibition of differentiation. Increased expression of C/EBPβ in both APL and chronic myeloid leukemia cells after treatment with ATRA or imatinib, respectively, was shown to promote differentiation of AML cell lines, whereas its KD in APL cells reduced the differentiation potential of these cells.^41^^,^^46^

In conclusion, our data reveal how RUNX1::RUNX1T1 drives deregulation of the HSPC proteome, changing hundreds of proteins, including downregulation of C/EBPβ in normal human primary cells. Consistent with this observation, although *CEBPB* is generally overexpressed in AML at the mRNA level, patients with t(8;21) have among the lowest *CEBPB* expression levels of patients with AML. Ectopic expression of C/EBPβ promoted proliferation of myeloid cells in human primary progenitors. Conversely, shRNA-mediated KD of C/EBPβ inhibited the growth of normal myeloid cells; however, it promoted the growth of cells expressing RUNX1::RUNX1T1. C/EBPβ influences human granulocytic differentiation and may act as a lineage discriminator in human hematopoietic cells.

Conflict-of-interest disclosure: The authors declare no competing financial interests.

## Supplementary Material

Supplemental Figures, Methods, and References
