# Supplementary material for: Expression of RUNX1::RUNX1T1 deregulates the proteome, induces C/EBPβ suppression, and blocks myeloid differentiation
Source: Blood Neoplasia. 2026 Jun 18;3(3):100259. doi: 10.1016/j.bneo.2026.100259 (PMC13443899; doi:10.1016/j.bneo.2026.100259)
Supplement: Supplementary file 1 [file BNEO_NEO-2026-001058-mmc1.pdf]

Supplementary Figures and Methods

Article Title: RUNX1::RUNX1T1-induced C/EBPβ suppression blocks myeloid differentiation

Supplementary Figures

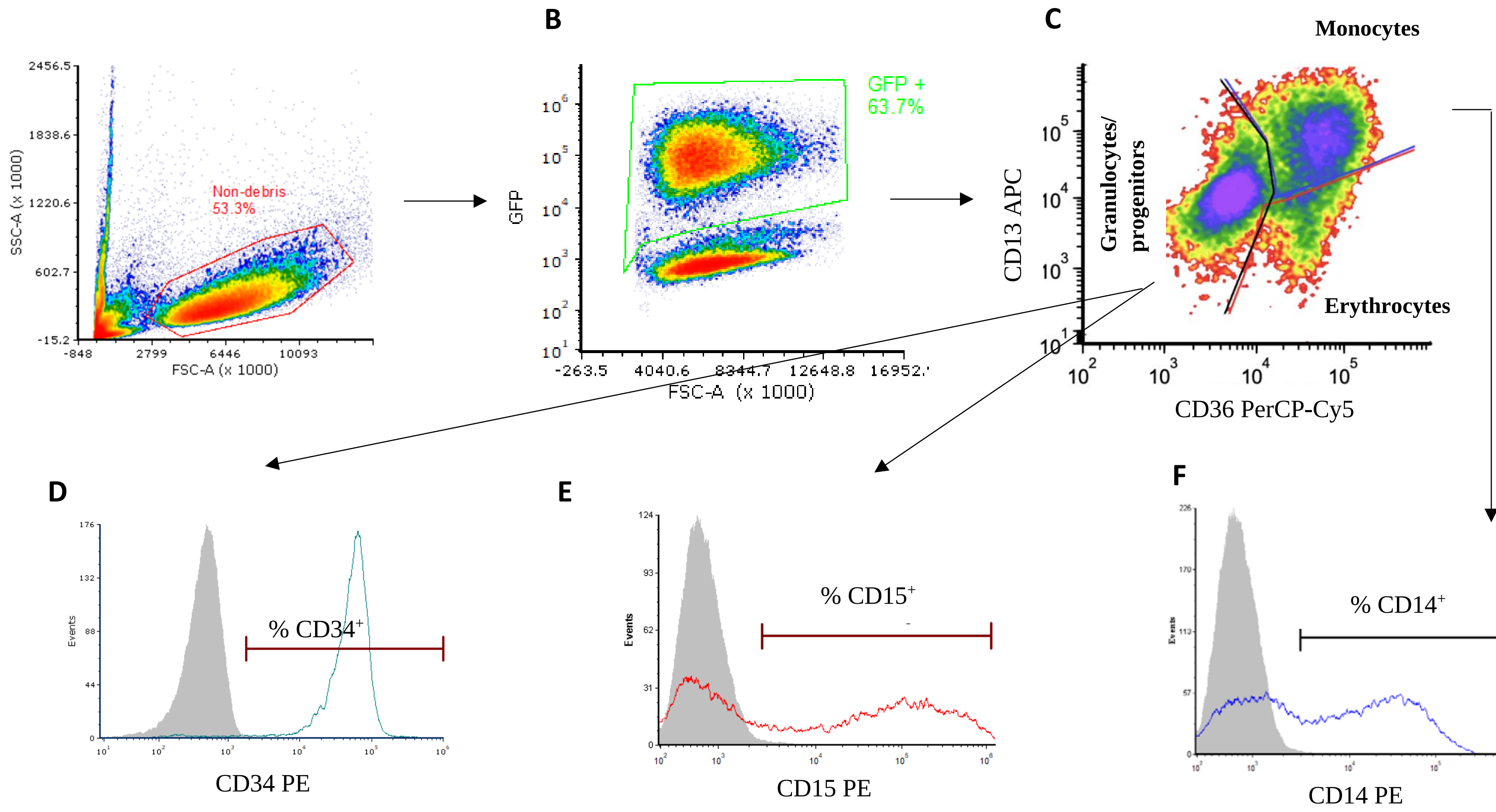

Supplementary Figure S1. Representative gating strategy used to immunophenotype HSPC

Representative density plots and histograms outlining the gating strategy used for the immunophenotypic analysis of HSPC over 17 days of culture. **A** Example gate used to exclude cell debris (non-debris), based on forward side scatter (FSC) and side scatter (SSC). **B** The percentage of GFP$^+$ cells within a bulk population was established based on the 'non-debris' gate shown in **A**. Background auto fluorescence was established using cells subjected to the equivalent viral infection procedure but in the absence of virus (mock culture). **C** CD13-APC and CD36-PerCP-Cy5 were used to discriminate between early progenitors/granulocytic (depending on day of analysis), monocytic and erythroid lineages within the GFP$^+$ population. **D-E** Each subpopulation was subsequently examined for differentiation markers, such as CD34$^+$, the granulocytic marker CD15 or the monocytic marker CD14 (using PE conjugated antibodies). Cells stained with the isotype control IgG (gray) were used to set the appropriate gating strategies.

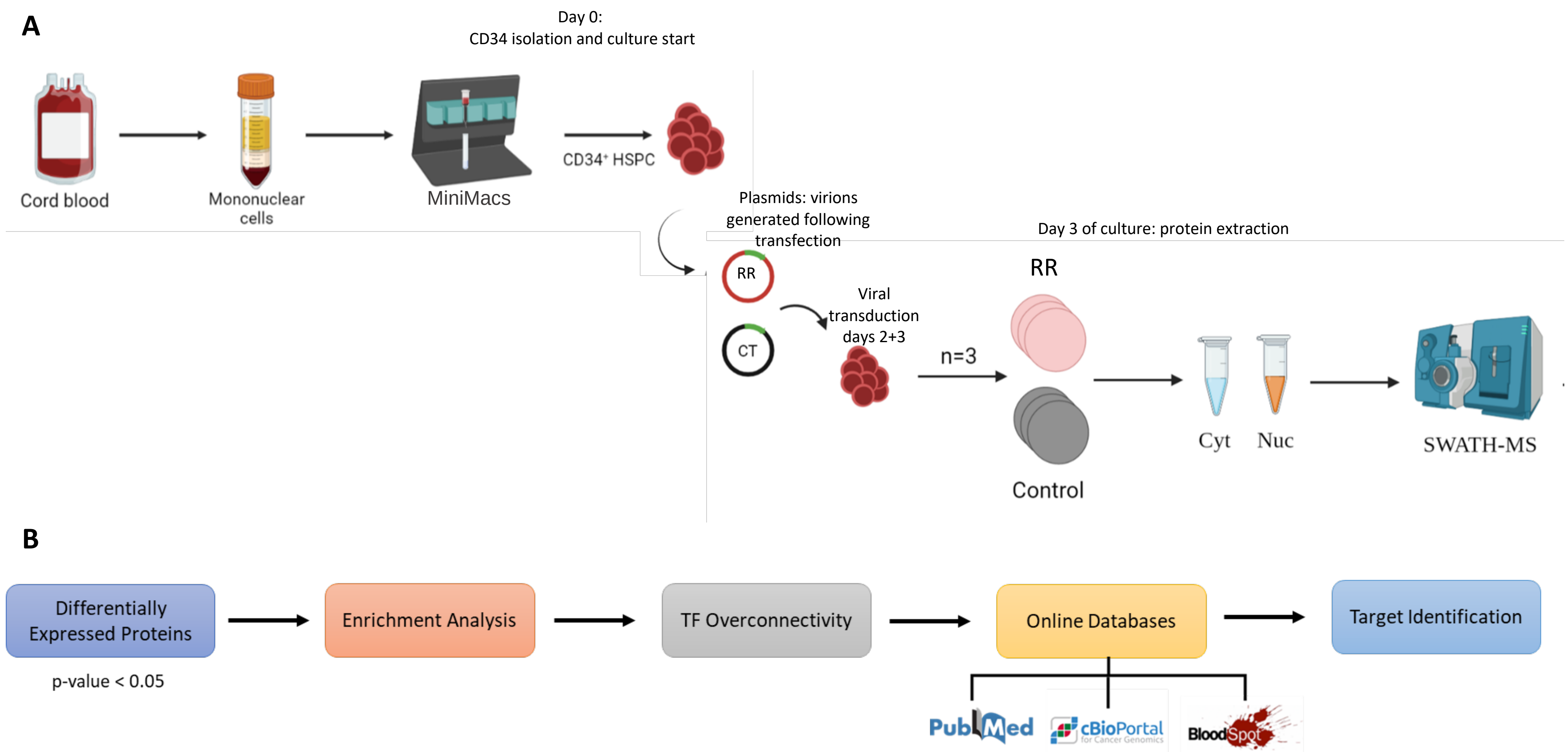

Supplementary Figure S2. Experimental design of the SWATH-MS analysis of proteomic changes associated with the expression of RUNX1::RUNX1T1 in HSPC

A Human cord blood derived CD34$^+$ HSPC were isolated using a MiniMacs^{TM} (Miltenyi Biotec) and infected with retrovirus to ectopically co-express RUNX1::RUNX1T1 and GFP (RR) or GFP alone (Control, CT) in three independent experiments. Following transduction on day 3 of culture, proteins were extracted from cytosolic or nuclear sub-cellular compartments and SWATH-MS was performed. **B** Statistically differentially expressed proteins were identified by applying a cut-off of p-value < 0.05. Subsequently, pathway analysis (enrichment analysis) followed by transcription factor (TF) overconnectivity was performed using Metacore^{TM}. To identify important TF for downstream analysis, literature search for novelty, coupled with mRNA expression in AML and normal hematopoiesis was performed using publicly available datasets. Figure created using Biorender.

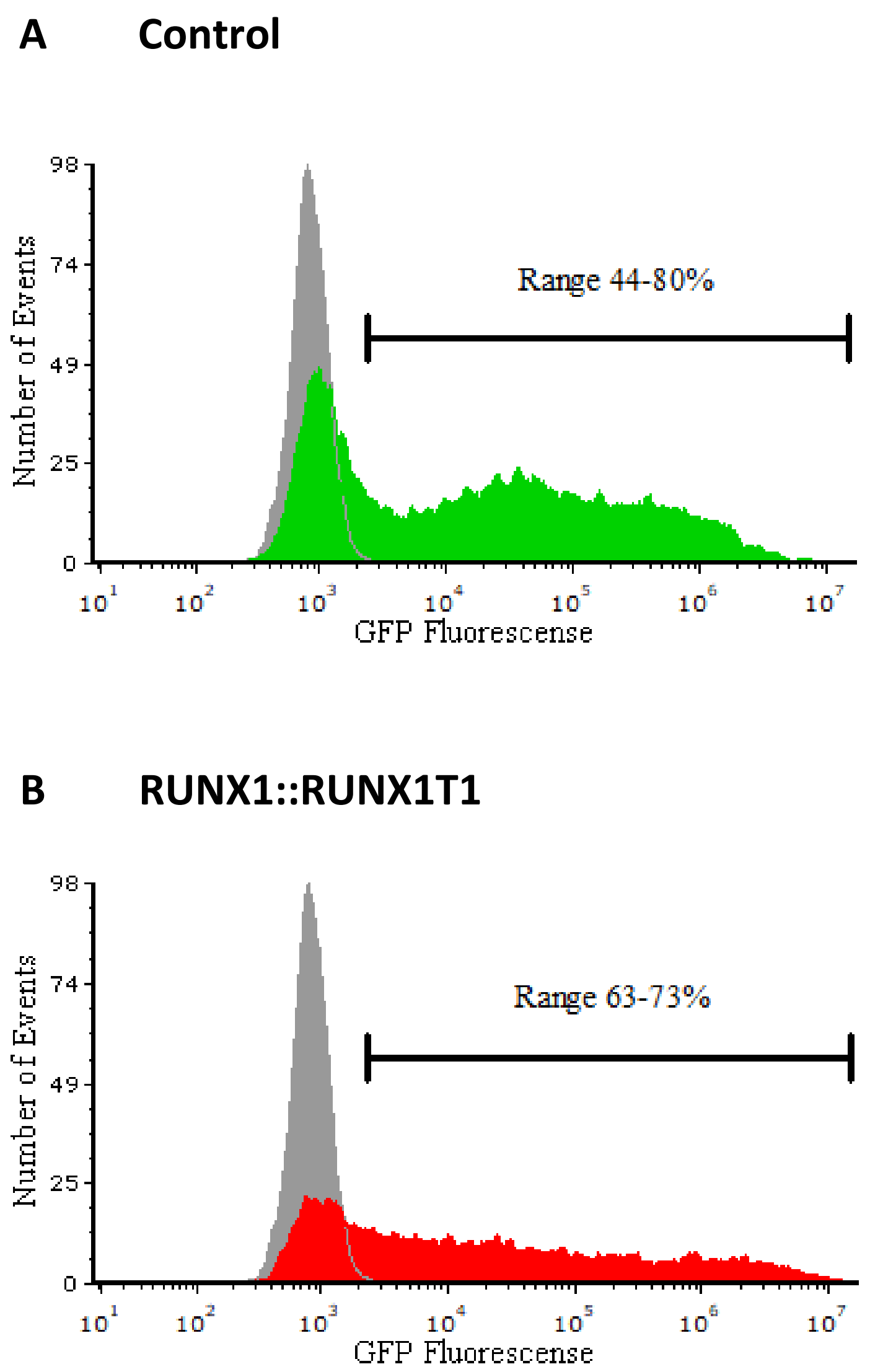

Supplementary Figure S3. GFP expression of infected CD34$^+$ HSPC expressing RUNX1::RUNX1T1

Representative flow cytometric histograms showing percentage green fluorescent protein (GFP) of CD34$^+$ HSPC in **A** Control and **B** RUNX1::RUNX1T1 transduced cells. Histograms were gated to exclude cell debris and dead cells based on FCS/SSC (as in Supplementary Figure S1). Background auto-fluorescence was established using HSPC subjected to the equivalent retroviral infection procedure but in the absence of retrovirus (mock) (gray). Percentage within the black marker line represents proportion of cells showing fluorescence greater than background autofluorescence at < 0.01% (n=3).

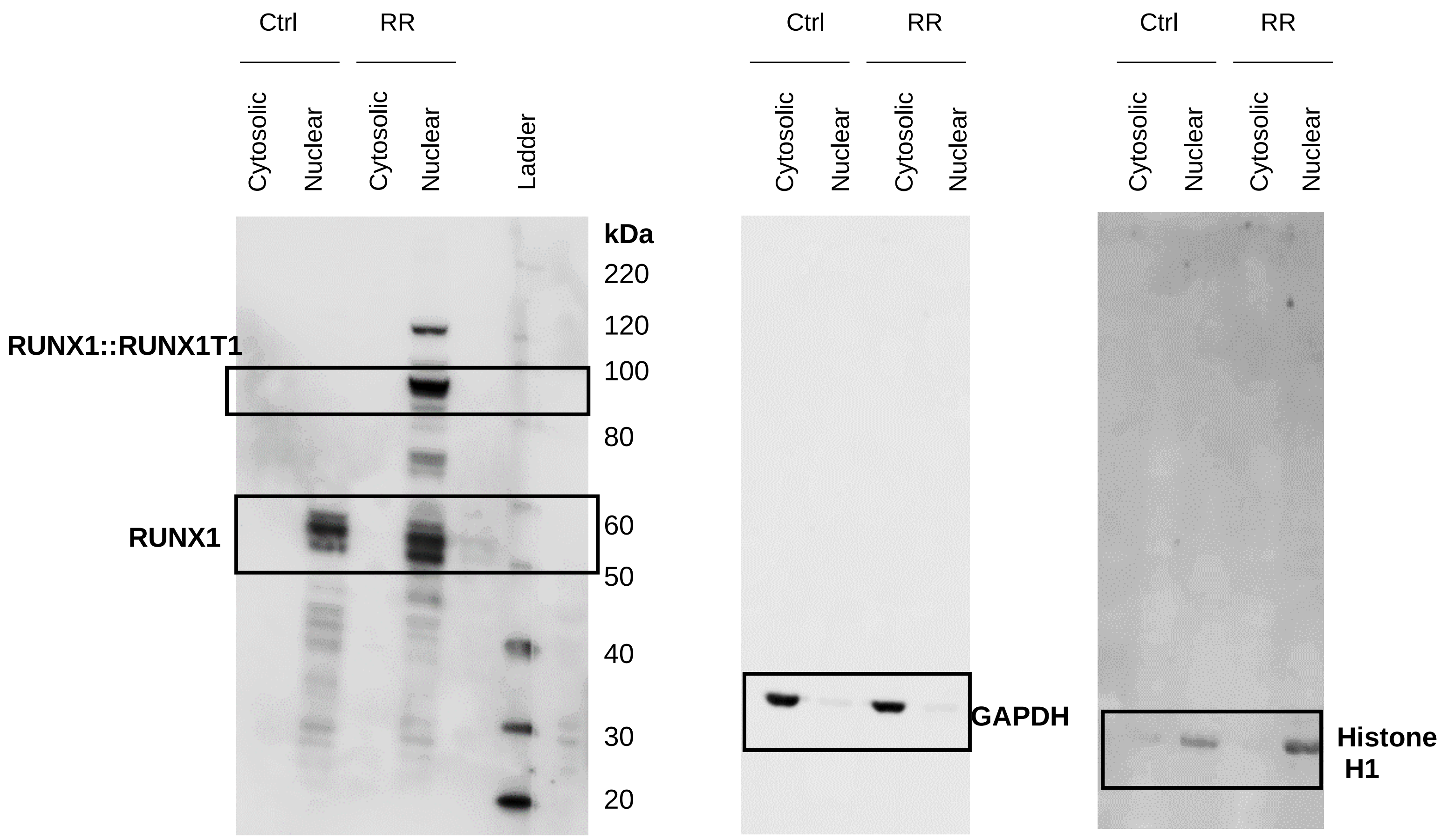

Supplementary Figure S4. Full length blots showing expression of RUNX1, GAPDH and Histone H1 in CD34+ HSPC transduced cells

Full length western blot showing protein expression of RUNX1, GAPDH and Histone H1 to accompany Figure 1. Anti-RUNX1 antibody was used to detect the N terminus (containing RUNX1) of the RUNX1::RUNX1T1 fusion protein. GAPDH and histone H1 were respectively used as loading controls and estimation of the subcellular fractionation efficiency for cytosolic and nuclear samples. Ctrl, control; RE, RUNX1::RUNX1T1.

A

B

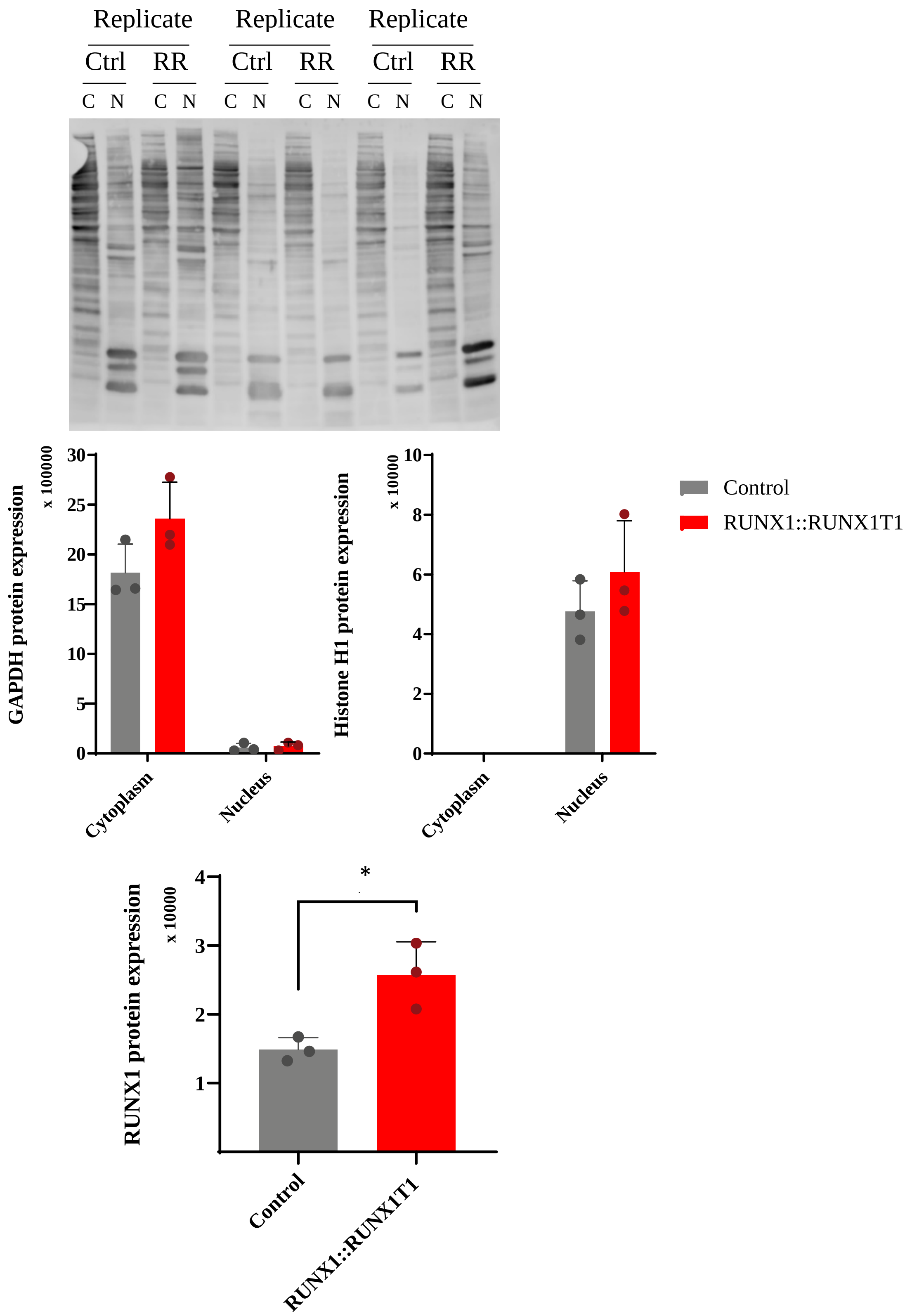

Supplementary Figure S5. Expression of RUNX1::RUNX1T1 in CD34⁺ HSPC transduced cells

A Qualitative assessment of cytoplasmic (C) and nuclear (N) fractionated protein samples was assessed following immunoblotting of 1 µg of protein sample extract. SDS-PAGE coupled with electroblotting onto PVDF membrane was initially performed followed by SYPRO™ Ruby Protein Blot Stain. Extracts from three independent experiments (replicates) used in SWATH-MS are shown. $CD34^+$ HPSC transduced with a viral vector encoding RUNX1::RUNX1T1 (RR) or control (Ctrl; GFP alone). **C** SWATH-MS analysis was performed on cytosolic and nucleus fractions of $CD34^+$ HSPC infected with

RUNX1::RUNX1T1 or control viruses on day 3 of cell culture. Quantitated proteins were filtered to include proteins detected in all biological replicates in both control and RUNX1::RUNX1T1 expressing cells for each subcellular compartment. Left Panel, detection of GAPDH and Right Panel, Histone H1 protein by SWATH-MS. Lower Panel, given that RUNX1::RUNX1T1 is a fusion protein formed through a chromosomal translocation, it is not detectable as a 'single protein' by SWATH-MS. However, RUNX1 levels may be used as an approximate indicator for RUNX1::RUNX1T1 levels (see discussion). SWATH-MS quantification of RUNX1 expression (or RUNX1 peptides) in the nucleus of $CD34^+$ HSPC transfected with a RUNX1::RUNX1T1 retroviral vector as compared to the nuclear compartment of control cells. Data indicates mean ±1SD (n=3). Significant differences between RUNX1::RUNX1T1 and control cells in lower panel was analyzed using paired t-test, * denotes $p<0.05$.

Protein	UniProtID	p-value	FC	Loc.	Metacore™		HSPC§	
					Interactome TF	p-Value	Reg.	FC
**NFKB2**	Q00653	3.30×10^{-4}	3.2	C	✗	-	n/a	
**TRIM28**	Q13263	7.16×10^{-4}	1.5	C	✗	-	n/a	
**SPI1**	P17947	1.11×10^{-3}	-1.9	N	✓	1.453×10^{-10}	↓	-1.76
**KMT2A**	Q03164	5.97×10^{-3}	1.6	N	✗	-	n/a	
**ZBTB48**	P10074	6.90×10^{-3}	1.4	N	✗	-	n/a	
**LRRFIP1**	Q32MZ4	7.01×10^{-3}	1.31	C	✗	-	n/a	
**EDF1**	O60869	1.10×10^{-3}	1.4	N	✗	-	n/a	
**CEBPβ**	P17676	1.17×10^{-2}	-2.1	N	✓	1.39×10^{-4}	↓	-1.6
**RB1**	P06400	1.54×10^{-2}	2.4	N	✗	-	n/a	
**RUNX1**	Q01196	2.08×10^{-2}	1.7	N	✓	1.53×10^{-32}	↑	1.91
**CBFβ**	Q13951	2.99×10^{-2}	1.4	N	✓	1.571×10^{-5}	No change	
**BAZ2A**	Q9UIF9	3.66×10^{-2}	1.4	N	✗	-	n/a	
**TTF2**	Q9UNY4	3.71×10^{-2}	-1.8	N	✗	-	n/a	
**HIRA**	P54198	3.80×10^{-2}	-1.4	N	✗	-	n/a	
**ZBTB49**	Q6ZSB9	3.89×10^{-2}	-1.2	N	✗	-	n/a	
**TSHZ1**	Q6ZSZ6	4.49×10^{-2}	1.6	C	✗	-	n/a	

Supplementary Figure S6. Identification of RUNX1::RUNX1T1 induced TF protein dysregulation in human HSPC

Statistically significant changing transcription factor (TF) proteins were determined by SWATH-MS analysis on cytosolic and nuclear extracts of human $CD34^+$ HSPC infected either with a RUNX1::RUNX1T1 retroviral vector, or a control plasmid, on day 3 of cell culture. ANOVA was used to determine significance of expression (P<0.05). Linear fold change in RUNX1::RUNX1T1 expressing cells compared to controls are shown. Green indicates upregulation in RUNX1:RUNX1T1 cells compared to control. Red (and negative sign) indicates down regulation in RUNX1::RUNX1T1 cells compared to control. To identify significantly overconnected TF within the dataset, the Metacore™' 'Interactome Transcription Factors' algorithm was used. ✓ and ✗ denotes if the gene was significantly identified or not (respectively) by the algorithm. §Normalized mRNA TF expression levels was compared between RUNX1::RUNX1T1 and control $CD34^+$ HSPC using microarray analysis, performed on day 3 of cell culture (using a dataset previously generated [1]). ↑ and ↓ respectively denotes mRNA upregulation or downregulation (negative FC values) of corresponding gene in RUNX1::RUNX1T1 HSPC, as compared to control. FC, fold change. C, change in cytoplasm; N, changed in nucleus. n/a, not applicable as changes were only examined in significantly identified TF using the TF interactome tool.

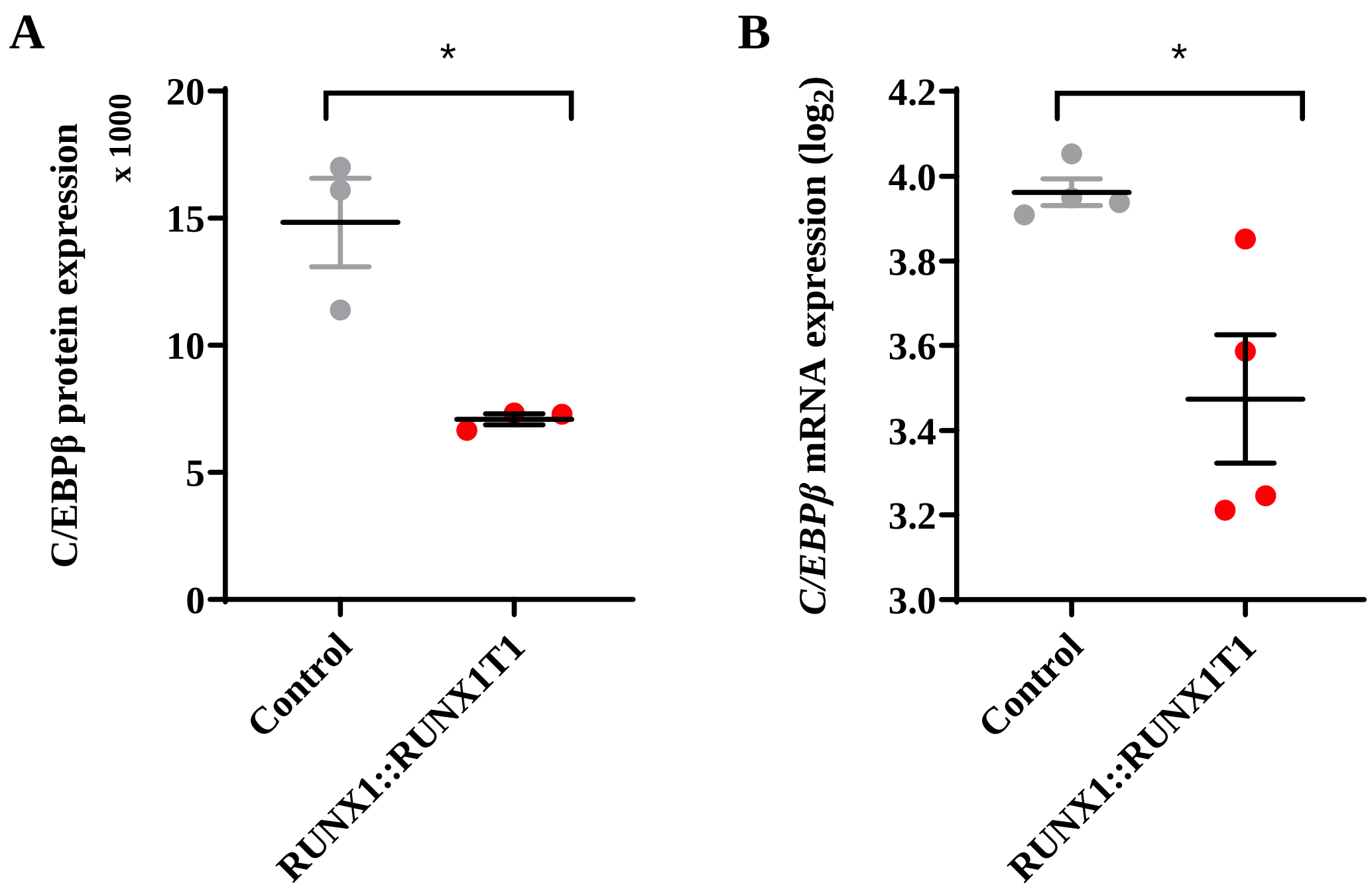

Supplementary Figure S7. CEBPβ expression profile in CD34⁺ HSPC expressing RUNX1::RUNX1T1

A C/EBPβ protein expression profile was obtained by performing SWATH-MS analysis on CD34⁺ HSPC transfected with either a control or a RUNX1::RUNX1T1. This protein was found to be expressed solely in the nucleus. Data represents mean ± 1SD (n=3). Significant difference between RUNX1::RUNX1T1 and control cells was analyzed using paired t-test; * denotes $p<0.05$. **B** Normalized microarray data from[1] (data set E-MEXP-583) showing $\log_2$ *CEBPB* mRNA expression in normal human HSPC expressing RUNX1::RUNX1T1 compared to control on day 3 of cell culture[1]. Data represents mean ± 1SD (n=4). Significant difference between RUNX1::RUNX1T1 and control cells was analyzed using paired t-test; * denotes $p<0.05$.

A

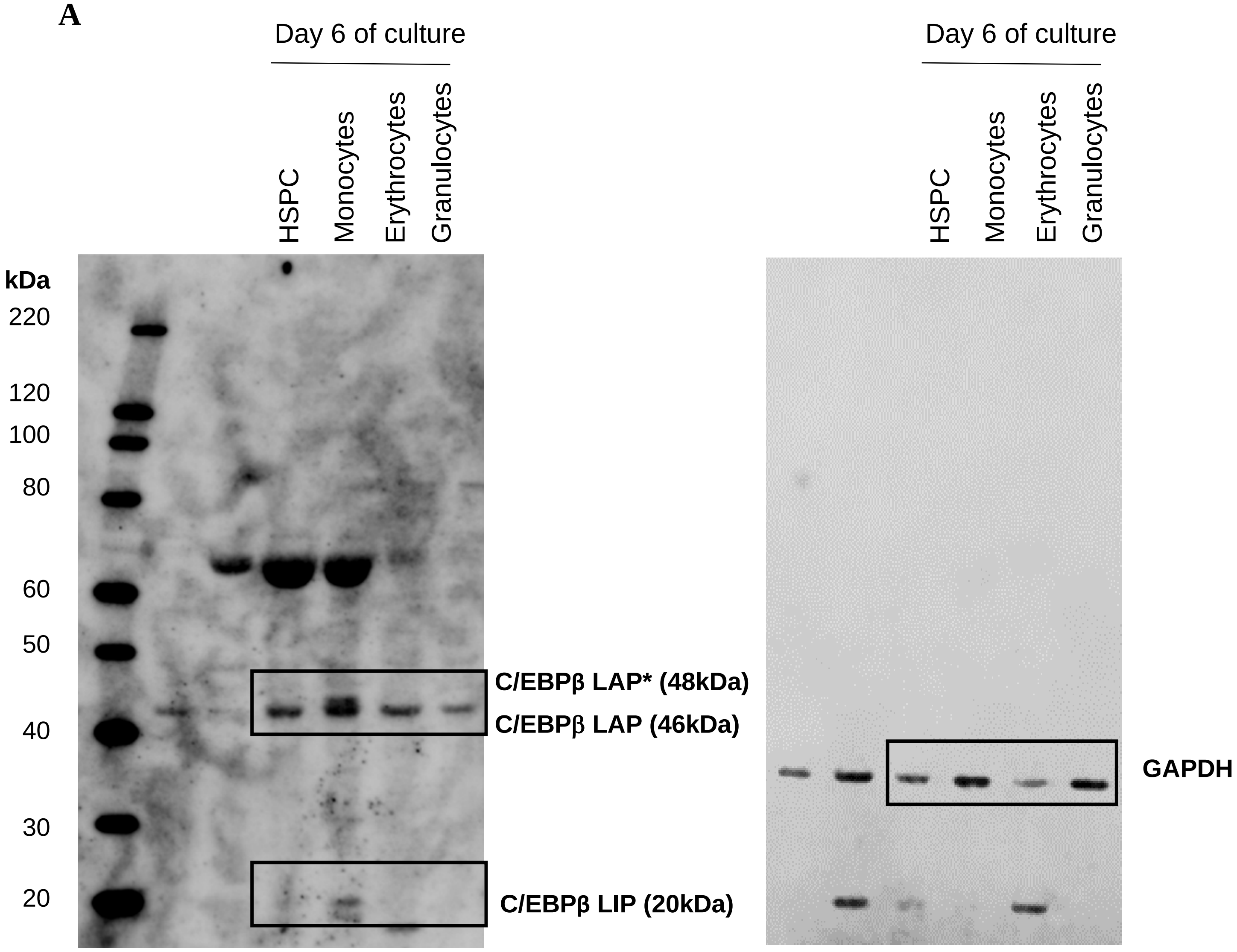
Day 6 of culture
HSPC
Monocytes
Erythrocytes
Granulocytes
kDa
220
120
100
80
60
50
40
30
20
C/EBPβ LAP* (48kDa)
C/EBPβ LAP (46kDa)
C/EBPβ LIP (20kDa)
Day 6 of culture
HSPC
Monocytes
Erythrocytes
Granulocytes
GAPDH

B

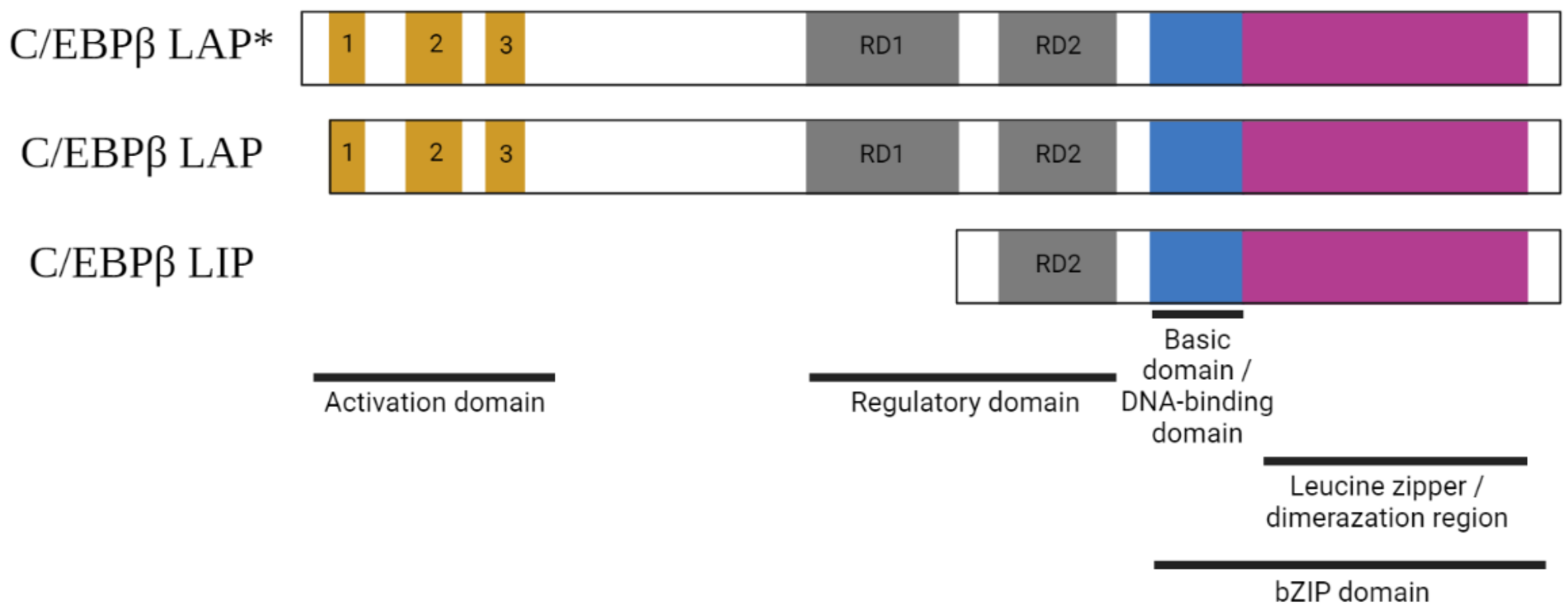
C/EBPβ LAP*
C/EBPβ LAP
C/EBPβ LIP
1
2
3
RD1
RD2
Activation domain
Regulatory domain
Basic domain / DNA-binding domain
Leucine zipper / dimerazation region
bZIP domain

Supplementary Figure S8. C/EBPβ protein expression in normal human $CD34^+$ HSPC and human hematopoietic lineages

A Full length western blot showing C/EBPβ total protein levels in normal human HSPC (total), and in monocytic ($CD14^{high}$); erythroid ($CD14^{low}CD36^{high}$) and granulocytic ($CD14^{low}CD36^{low}$) progenitor cells isolated from culture during differentiation of $CD34^+$ HSPC into hematopoietic lineages (day 6 of culture in IL-3, SCF, G/GM-CSF) as previously described[1]. GAPDH was used as a loading control. **B** Schematic representation of the three C/EBPβ protein isoforms and their functional domains. Liver-enriched activating protein* (LAP* or full-length [FL]), liver-enriched activating protein (LAP) and liver-enriched inhibitory protein (LIP). LAP* and LAP isoforms contain three transactivation domains, as well as two regulatory regions RD1 and RD2. LIP, on the other hand, lacks the transactivation domain, and only contains the regulatory region RD2. All three isoforms possess a DNA binding domain, couples with a leucine zipper region, making up the bZIP domain. Note unlabelled lanes are not related to this study but have been left in, so the molecular weight ladder remains, so readers can judge protein kDa.

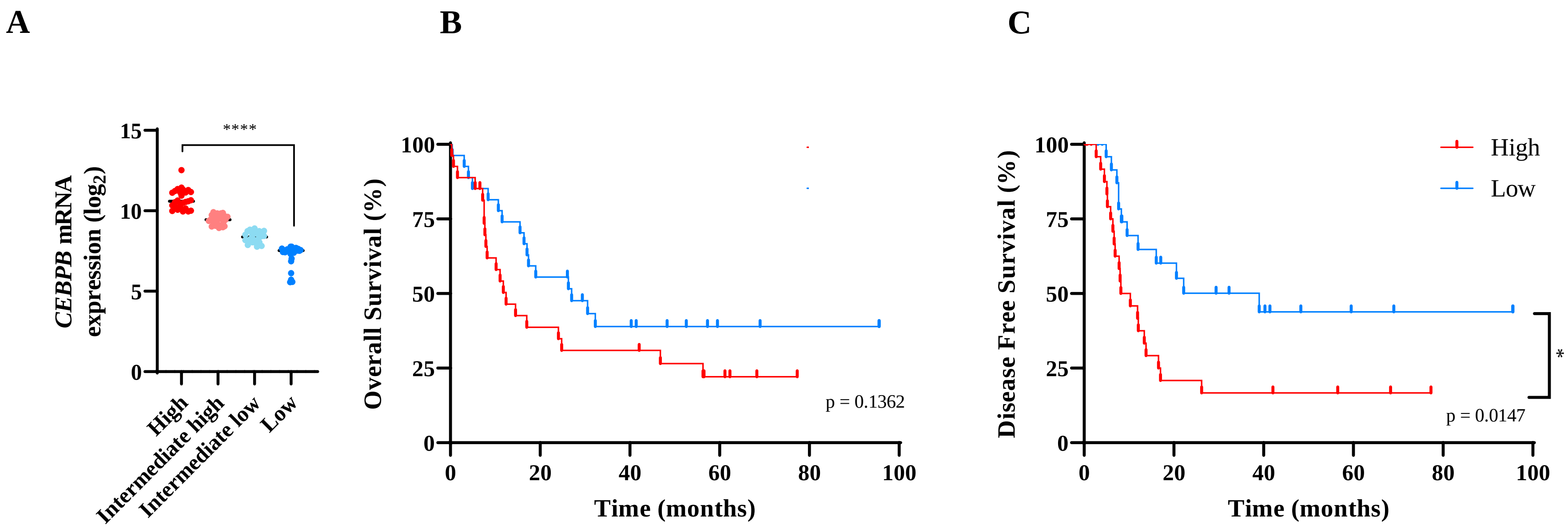

CEBPB expression	Number of patients	Median OS (months)	Hazard ratio	95% CI
High	27	12.4	1.6	0.8-3.2
Low	27	27		

CEBPB expression	Number of patients	Median DSF (months)	Hazard ratio	95% CI
High	27	9.25	2.4	1.2-4.8
Low	27	39		

Supplementary Figure S9. High level of *CEBPB* is associated with poorer disease-free survival of AML patients

A Normalized (log_2) *CEBPB* mRNA expression of AML data obtained from TCGA[2] using cBioPortal (www.cbioportal.org). AML patients were stratified according to quartiles: upper quartile (high, n=27) and lower quartiles (low, n=27). The intermediate quartiles were not used in the subsequent analysis. Significance determined by one-way ANOVA; **** denotes $p<0.0001$. **B** Overall survival and **C** disease-free survival analysis of AML patients stratified according to upper (high) and lower (low) *CEBPB* mRNA expression quartiles. Untreated patients and t(15;17) AML patients (that present a different treatment regime compared to other AML subtypes) were excluded from this analysis. Statistical analysis was performed using the Log-Rank test between high and low *CEBPB* expression groups.

A

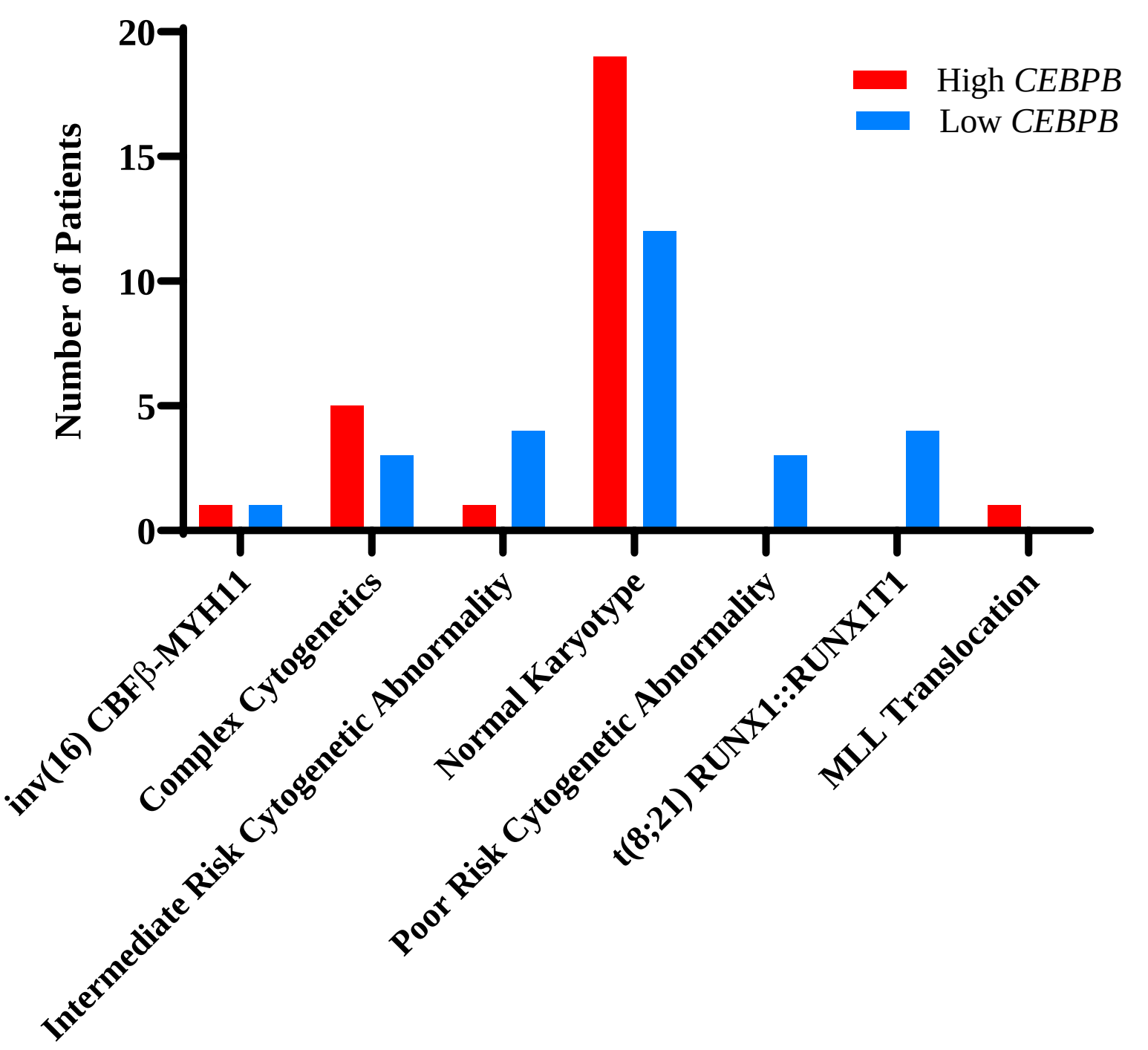

B

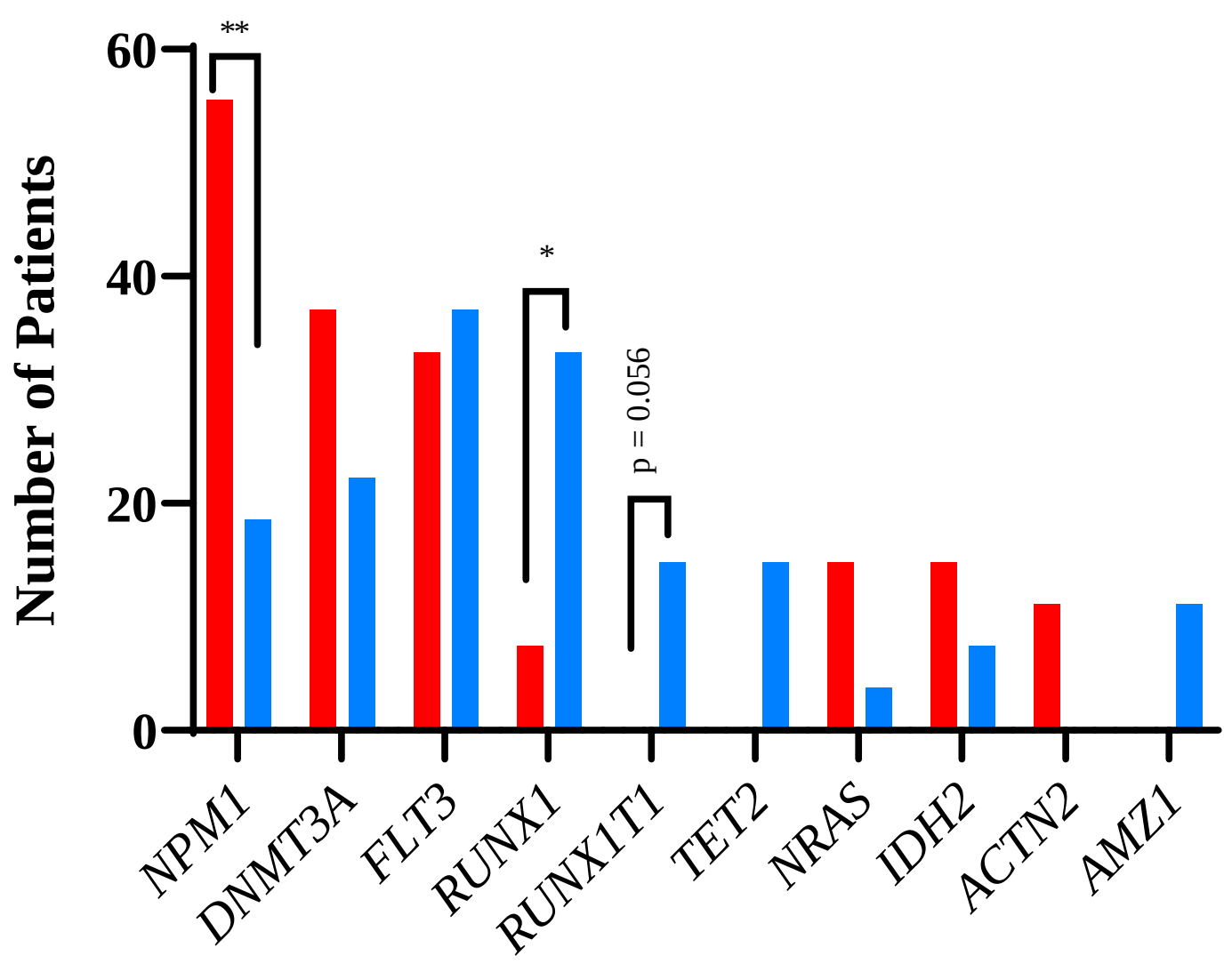

Supplementary Figure S10. Lower levels of *CEBPB* expression correlates with t(8;21) and mutations in RUNX1 in AML patients

A Relationship between high and low *CEBPB* mRNA expression and cytogenetic abnormalities of AML patients. **B** Relationship between increased and reduced *CEBPB* mRNA expression and the occurrence of specific molecular abnormalities in AML patients, according to *CEBPB* high (n=27) and low (n=27) quartiles. Data obtained from TCGA [2] using cBioPortal (www.cbioportal.org). Statistical analysis was performed using one-sided Fisher Exact test. * denotes p<0.05; ** denotes p<0.01.

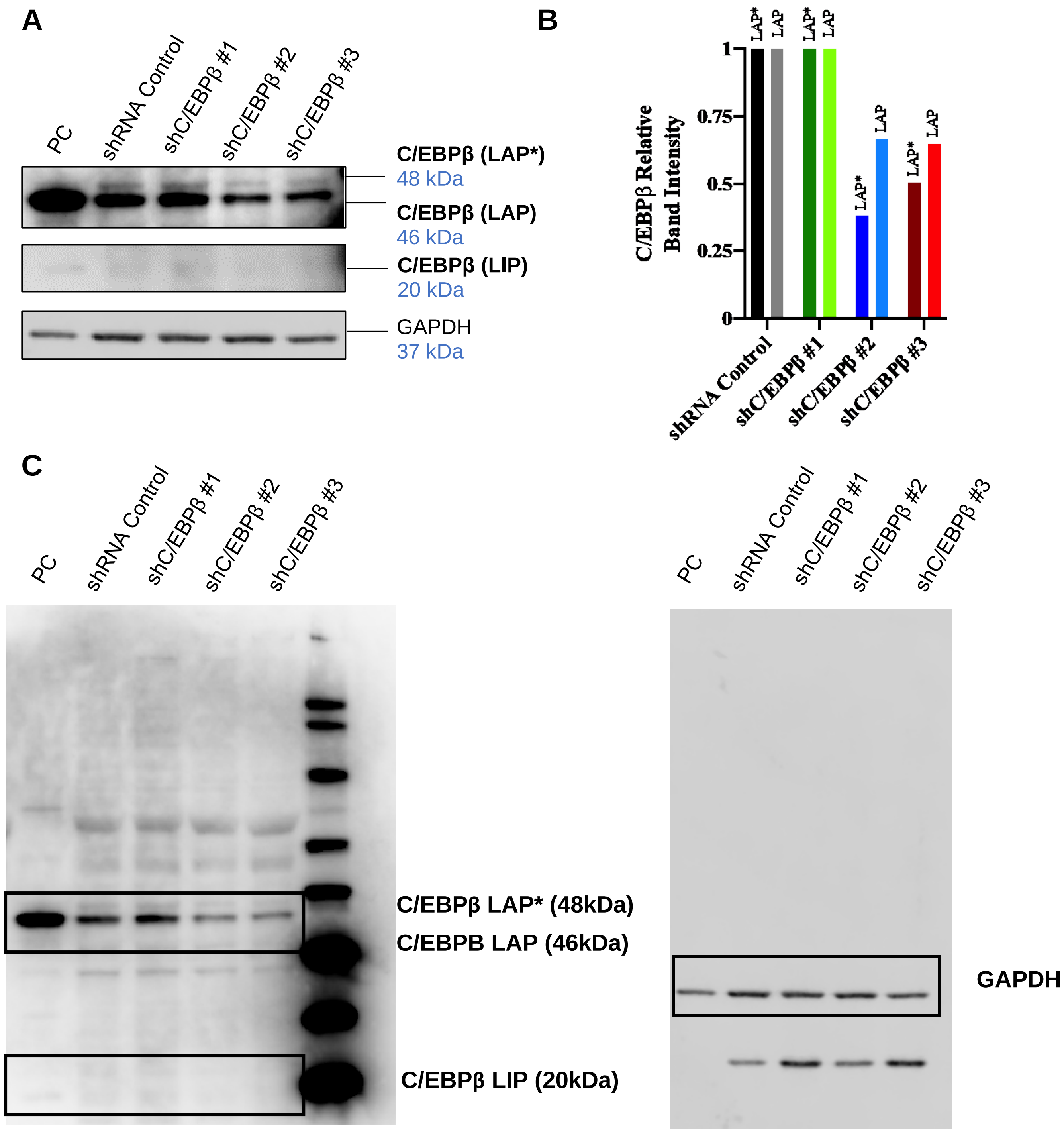

Supplementary Figure S11. C/EBPβ knockdown in human HSPC

A Western blot analysis showing C/EBPβ protein expression in green fluorescent (GFP⁺) hematopoietic stem progenitor cells (HSPC) transduced with a control or C/EBPβ-targeted shRNA constructs, sorted for GFP⁺ on day 6 of culture. HEK 293T transfected with a C/EBPβ-overexpression construct was used as a positive control (PC); GAPDH was used as a loading control. Full blot depicted in C. **B** Bar chart showing densitometry analysis regarding C/EBPβ expression in HSPC transduced with three shC/EBPβ constructs. C/EBPβ expression was quantified using ImageJ, normalized to loading control (GAPDH) and to shRNA control (n=1). **C** Full length western blot showing C/EBPβ expression in HSPC transduced with a control or C/EBPβ-targeted shRNA constructs, on day 6 of culture.

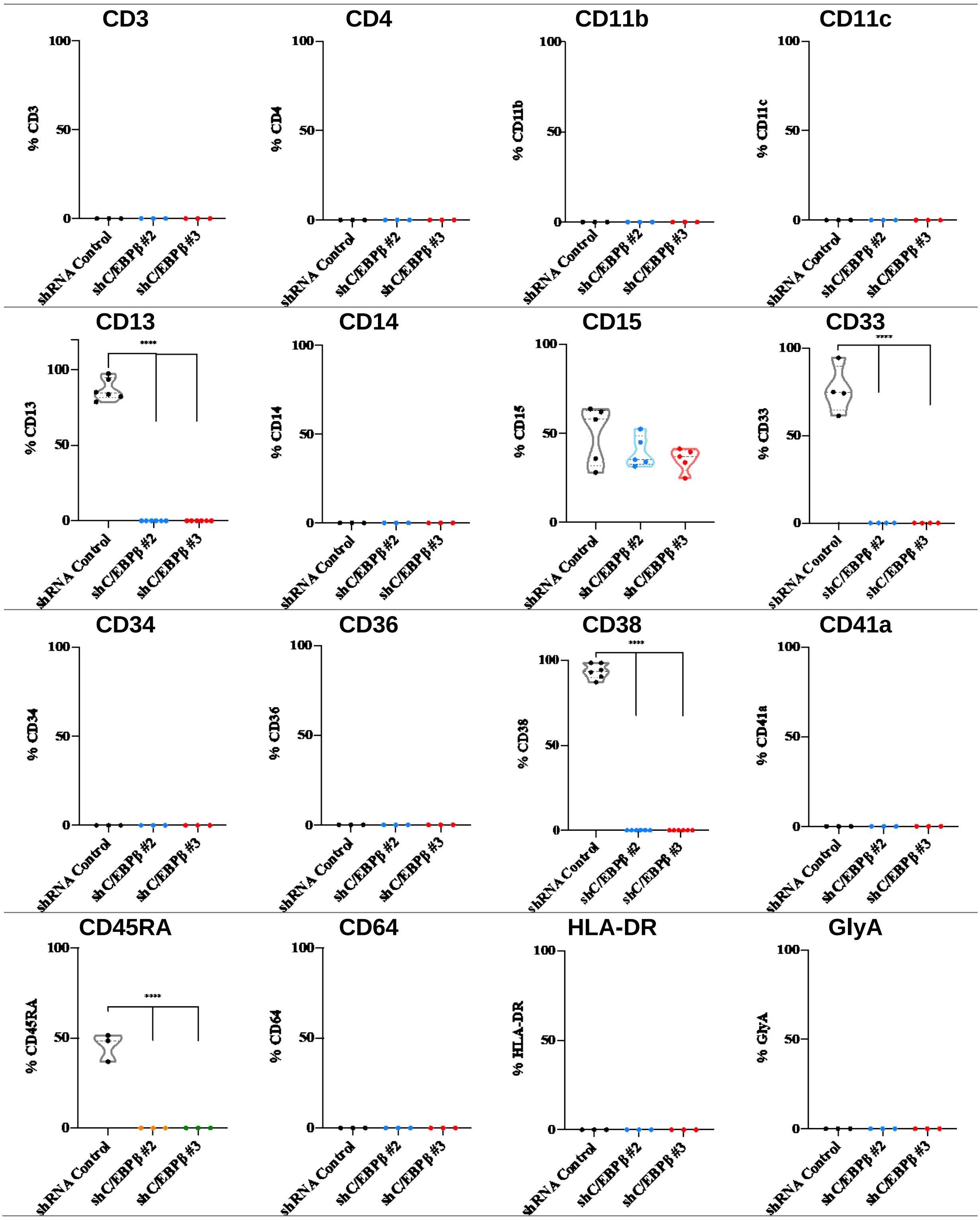

Supplementary Figure S12. The effect of C/EBPβ KD on cell surface marker expression in SKNO-1 cells.

Expression of cell surface markers in SKNO-1 cells transduced with a control or shC/EBPβ constructs. An isotype control (IgG) was used to define negative marker expression (n≥3) Significant difference between shC/EBPβ cultures and control were analyzed using one-way ANOVA with Bonferroni's multiple comparisons correction; **** $p<0.0001$.

A

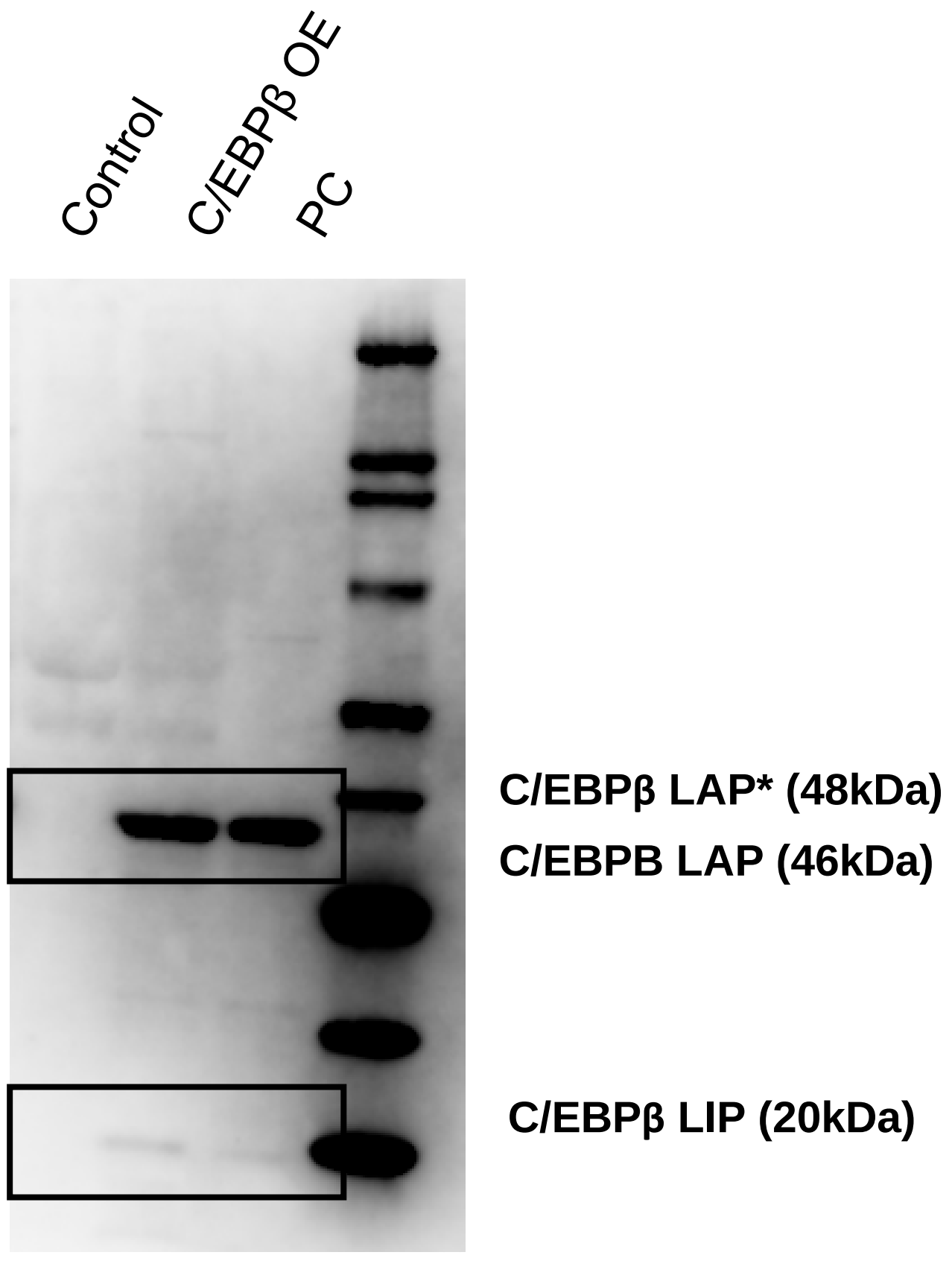

B

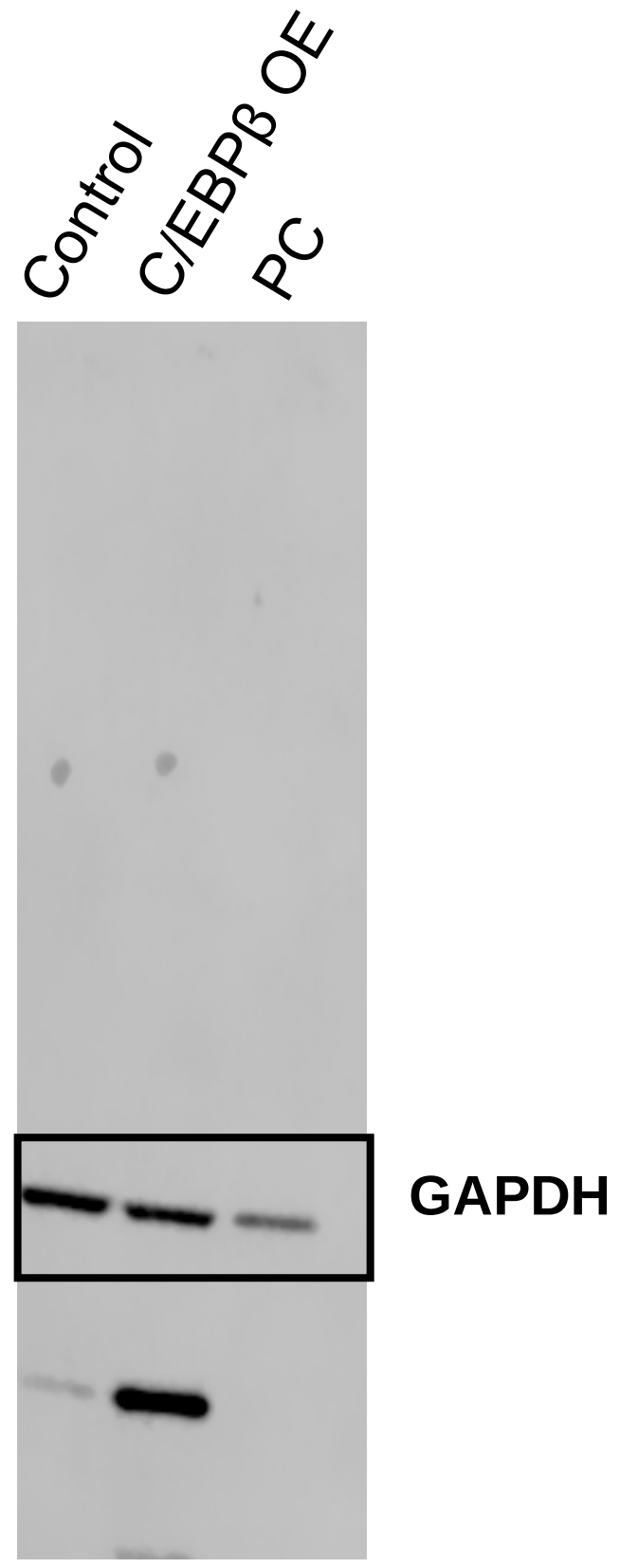

Supplemental Figure S13. C/EBPβ overexpression in human CD34+ HSPC

A Western blot analysis showing C/EBPβ protein expression in FACS HSPC transduced with a control or a C/EBPβ LAP* overexpression construct. HEK 293T transfected with a C/EBPβ-overexpression construct was used as a positive control (PC). **B** GAPDH was used as a loading control.

Supplementary Methods

Plasmids and generation of retro- and lentivirus

Human primary and acute myeloid leukemia (AML) cell lines were transduced with amphotropic retro- or lentiviruses derived from the following plasmids:-

Plasmid	Gene Target	Marker	Vector name	Source
PINCO	RUNX1::RUNX1T1	GFP	RUNX1::RUNX1T1	[3,4]
PINCO	Non-coding Stuffer fragment	GFP	Control	[3,4]
pLV	C/EBPβ NM_005194.3	Puro; GFP	C/EBPβ OE	VectorBuilder VB900000-7325epv
pLV	Empty Vector	Puro; GFP	Control	VectorBuilder VB160608-1061ubk
pLV	C/EBPβ shRNA #1 TRCN0000007440	Puro; GFP	shC/EBPβ #1	VectorBuilder VB900036-5734ysy
pLV	C/EBPβ shRNA #2 TRCN0000364533	Puro; GFP	shC/EBPβ #2	VectorBuilder VB900037-4803rzb
pLV	C/EBPβ shRNA #3 TRCN0000364495	Puro; GFP	shC/EBPβ #3	VectorBuilder VB900036-5748zjr
pLV	Scrambled shRNA	Puro; GFP	shRNA Control	VectorBuilder VB180406-1061anb
pMD2	Envelope plasmid	n.a	Envelope	Addgene
psPAX2	Packaging plasmid	n.a	Packaging	Addgene

Puro – Puromycin; GFP – Green Fluorescent Protein; NM - National Center for Biotechnology Information (NCBI) reference sequence (RefSeq) database transcript accession number; TRCN – RNAi consortium shRNA Clone ID number.

For quantitating changes in the proteome using sequential window acquisition all theoretical mass spectra (SWATH-MS), a retroviral PINCO plasmid co-expressing RUNX1::RUNX1T1 cDNA and green fluorescent protein (GFP), or GFP alone were used to transduce $CD34^+$ hematopoietic stem progenitor cells (HSPC). Lentiviral (pLV) vectors purchased from VectorBuilder (Edinburgh, U.K.) co-expressing CEBPB cDNA and GFP were used in overexpression studies whilst short-hairpin RNA (shRNA) co-expressing GFP and puromycin selectable markers were used for knockdown (KD) studies.

Lipofectamine 3000 (Fisher Scientific, Loughborough, U.K.) was used to generate lentivirus using the viral packaging cell line, HEK 293T, according to the

manufacturer's instructions. Calcium phosphate transfection (Invitrogen, Paisley U.K.) was used to generate retroviral particles using the Phoenix packaging cell line, as previously described[3].

Culture of AML cell lines

The origin and culture conditions of cell lines used in this study are outlined below.

Cell line	***Base culture medium***	***Description***	***FAB***	***Molecular characteristics***	***Source***
HEL	RPMI 1640 (10% FBS)	Erythroleukemia	M6	JAK2 mutation (V617F)	ECACC
SKNO-1	RPMI 1640 (10% FBS) 10 ng/mL GM-CSF	AML	M2	t(8;21) (q22;q22) – RUNX1::RUNX1T1	DSMZ
U937	RPMI 1640 (10% FBS)	Histiocytic Lymphoma	M5	t(10;11)(p12;q14) – MLLT10::PICALM	ATCC

ATCC – American Type Culture Collection (Virginia, USA); DSMZ - German Collection of Microorganisms and Cell Cultures (Braunschweig Germany); ECACC™ - European Collection of Authenticated Cell Cultures (Salisbury, U.K); RPMI - Roswell Park Memorial Institute-1640 Medium; GM-CSF- Granulocyte / Macrophage colony stimulating factor.

All lines were maintained in the appropriate culture media (see above) supplemented with 10% (*v/v*) heat-inactivated fetal bovine serum (FBS; 77133, Labtech International Ltd, Sussex, U.K.), 1% (*v/v*) L-glutamine (Invitrogen) and 20 µg/mL gentamycin (Life Technologies, Paisley U.K.), unless otherwise specified. Cells were cultured at 37°C in a humidified incubator (5% CO_2) and maintained in log phase growth. Genetic identity of the cell lines was confirmed by short tandem repeat (STR) analysis and mycoplasma contamination was monitored using MycoAlert detection kit (Sigma-Aldrich, Dorset, U.K.). Cells at passages greater than twenty were not used in the experiments performed in this study.

Isolation, culture, and transduction of human HSPC

Mononuclear cells (MNC) were isolated from human cord blood by density gradient centrifugation using Ficoll-Paque™(GE Healthcare, Little Chalfont, U.K.) as previously described[3]. $CD34^+$ HSPC were isolated from MNC by MiniMACS® Indirect CD34 microbead kit (Miltenyi Biotec, Woking, U.K.) according to the manufacturer's instructions. Following isolation, human $CD34^+$ HSPC were cultured in Iscove's Modified Dulbecco's Medium (IMDM) supplemented with 5 ng/mL of Interleukin (IL)-3, stem cell factor (SCF), granulocyte-colony stimulating factor (G-CSF), and granulocyte/monocyte (GM)-CSF (BioLegend, London, U.K.). Cells were sub-cultured every 2-3 days and seeded at different densities according to developmental stages.

Human $CD34^+$ HSPC were transduced with retro- or lentivirus as previously described[3]. Briefly, for overexpression and knockdown studies, appropriate tissue culture plates were coated with Retronectin® (30 µg/mL; Takara Biotech Inc., Shiga, Japan) prior to transduction. The following day, viral supernatants were added and centrifuged for 120 minutes at 2200 x g at room temperature (RT). Following centrifugation, viral supernatants were removed and $CD34^+$ HSPC were added to the wells. For overexpression studies, the process was repeated the following day, in which $CD34^+$ HSPC were lifted from the plate, and viral supernatant added for subsequent centrifugation under the same conditions. For KD studies a second viral coating step was not performed due to high viral titre.

Hematopoietic differentiation and morphological analysis

To assess myeloid cell differentiation, immunostaining coupled with flow cytometry (see below) was performed over time using a panel of cell surface

markers: CD13-APC and CD36-biotin/streptavidin PerCP-Cy™5.5 were used for lineage discrimination. Differentiation markers using PE-conjugated antibodies were used to assess differentiation status; CD11b, CD14, CD15 and CD34[5]. Cells were analyzed on an Accuri™C6 Plus cytometer (BD Biosciences) using the gating strategy outlined in *Online Supplementary Figure S1*.

**Antibody**	**Clone**	**Supplier**	**RRID**
Anti-Human CD11b-PE	ICRF44	BioLegend, London, UK	AB_314158
Anti-Human CD13-APC	WM15	BioLegend, London, UK	AB_314182
Anti-Human CD14-PE	HCD14	BioLegend, London, UK	AB_830679
Anti-Human CD15-PE	W6D3	BioLegend, London, UK	AB_756012
Anti-Human CD34-PE	581	BioLegend, London, UK	Not defined
Anti-Human CD36-Biotin	SMO	Ancell, U.K.	Not defined
Mouse IgG1-PE	MOPC-21	BioLegend, London, UK	AB_2847829
PerCP-Cy™5.5 Streptavidin	NA	BD Biosciences, Wokingham, UK	AB_2868934
Anti-Human CD3-APC	BW164/56	Miltenyi Biotec, UK	AB_244230
Anti-Human CD4-APC	M-T466	Miltenyi Biotec, UK	AB_871690
Anti-Human CD11b-APC	ICRF44	BioLegend, London, UK	AB_314162
Anti-Human CD11c-APC	3.9	BioLegend, London, UK	AB_493023
Anti-Human CD14-APC	HCD14	BioLegend, London, UK	AB_830861
Anti-Human CD15-PerCP	W6D3	BioLegend, London, UK	AB_893256
Anti-Human CD33-APC	P67.6	BioLegend, London, UK	AB_2565754
Anti-Human CD34-APC	581	BioLegend, London, UK	AB_1877154
Anti-Human CD36-APC	HM36	BioLegend, London, UK	AB_571994
Anti-Human CD38-APC	HIT2	BioLegend, London, UK	AB_314362
Anti-Human CD41a-APC	REA386	Miltenyi Biotec, UK	AB_2921967
Anti-Human CD45RA-APC	HI100	BioLegend, London, UK	AB_314415
Anti-Human CD64-APC	10.1	BioLegend, London, UK	AB_1595428
Anti-Human HLA-DR-PerCP	L243	BioLegend, London, UK	AB_893575
Anti-Human GlyA-PerCP	HI264	BioLegend, London, UK	AB_2562706

NA – Not applicable; Cambridge Bio – Cambridge Biosciences;

Assessment of HSPC Cell Morphology

On day 13-17 of culture, approximately 30,000 cells were transferred to a pre-assembled cytospin slide chamber with a glass slide and centrifuged at 60 x g for 5 min, using a Cytospin 3 (Fisher Scientific). Slides were stained with May-Grünwald-Giemsa and scanned using Zeiss AxioScan Z1 Slide Scanner (Carl Zeiss, New York, USA) at 20x magnification. Differential counts were performed using QuPath (Edinburgh, U.K.). Cells were defined as being in an early, intermediate, or late phase of development if they were presented as myeloblasts/promyelocytes, myelocytes/metamyelocytes and band/segmented cells, respectively.

Determination of cell growth

Cells in log phase were counted and seeded at a concentration of $1x10^5$ cells/mL in a 24-well culture plate (1mL). Following seeding, 50 µL of cell suspension was recovered and transferred to flow cytometric tubes to confirm cell count. In addition, 100 µM TOPRO-3 was added to each flow cytometric tube to assay cell viability during cell culture. Viable cell counts were performed on indicated days and cumulative fold expansion was plotted over time.

Apoptosis

Apoptosis was determined using the Annexin V-APC Apoptosis Kit (ThermoFisher), according to the manufacturer's instructions. Briefly, washed cells were incubated with Annexin V-APC, following by the addition of propidium iodide (PI) (50 µg/mL) or 7-AAD (10 µg/mL). Samples were analyzed by flow cytometry.

Cell Cycle

To determine changes in cell cycle, PI (40 µg/mL) was used to label intracellular DNA. Briefly, cells fixed overnight using 70% (*v/v*) ethanol, followed by washing and staining with PI; containing 100 µg/mL RNase (Sigma-Aldrich) diluted in

1x PBS, at 37°C for 30 min. Samples were acquired within 20 min of incubation. Cell cycle analysis was performed using FCS Express (v6, DeNovo Software, California, USA) Multicycle AV DNA analysis tool plug-in.

Flow cytometry

Data was acquired using an Accuri C6 Plus Flow Cytometer™ (Accuri Cytometers, BD) and analyzed using FCS Express® v6 software (De Novo Software). Background auto fluorescence was established using cells subjected to the equivalent viral infection procedure but in the absence of virus (mock culture). For cell surface staining, background fluorescence was established using isotype controls. At least 10,000 events were recorded for each sample at a medium flow rate.

Western Blotting

To generate fractionated protein cell lysates, extractions were performed using the Biovision Nuclear/Cytosol Fractionation Kit (Cambridge Bioscience, Cambridge, U.K.) following manufacturer's instructions. Protein quantification was performed using Bradfords reagent solution (Sigma Aldrich) and measuring absorbance at 595 nm.

Protein electrophoresis was performed using the NuPAGE electrophoresis system, as described in[3]. The antibodies used in this study are described below.

Antibody	***Cat. #***	***Species***	***Conjugate***	***Dilution***	***Source***	***RRID***
AML1 (RUNX1)	#4334	Rabbit	-	1:5000	CST	AB_2184099
C/EBPβ	#90081	Rabbit	-	1:1000	CST	Not defined
Anti-rabbit	NA934	Donkey	HRP	1:10000	GE Healthcare	AB_772206
GAPDH DyLight® 680	GA1R	Mouse	DyLight® 680	1:10000	ThermoFisher	AB_2537657
Histone H1AF ® 790	AE-4	Mouse	AF790	1:5000	SCBT	AB_675641

AF90 – Alexa Fluor 790; **CST** – Cell Signaling Technologies (London, UK); **HRP** – Horseradish peroxidase; **O/N** – Overnight; **RRID** – Research Resource Identifier; **SCBT** – Santa Cruz Biotechnology (Heidelberg, Germany).

Equal protein loading was assessed using GAPDH or Histone H1 antibodies for total/cytosolic and nuclear protein extracts, respectively. HRP-conjugated secondary antibodies were purchased from GE Healthcare, U.K. and chemiluminescence detected using ECL prime (GE Healthcare).

Changes in protein expression were semi-quantitatively measured using Image J software (Fiji; v.2.0; U.S. National Institutes of Health, Maryland, USA). Briefly, a region of interest was drawn around a band of interest. From this, a histogram of peak intensity was generated, and a baseline of background intensity was set from the area surrounding the band within the region. Subsequently, the area under the curve was calculated to give an arbitrary intensity value. The band intensity of the protein of interest was normalized to the band intensity of the loading control for each sample. Furthermore, to compare protein expression between a test sample and the corresponding control, the first was normalized to the latter.

SYPRO™ Ruby Protein Blot Stain

Following electroblotting, membranes were stained with SYPRO® Ruby protein blot stain according to the manufacturer's instructions (ThermoFisher Scientific). Briefly, membranes were incubated with the stain for 15 min in a rotary shaker. Following incubation, gels were washed 4-6 times with water for 1 min and visualized in an Odyssey® Fc Imaging System (LI-COR Biosciences, Cambridge, U.K.).

SWATH-MS

Sample preparation was performed at Cardiff University and sample processing was performed at the Stoller Biomarker Discovery Centre at the University of Manchester by Dr. Andrew Pierce and Dr. Bethany Geary. Human cord

blood derived $CD34^+$ HSPC were isolated and transduced as described above (n=3) on day 3 of culture. Samples were prepared by diluting 10 µg of protein extract in ammonium bicarbonate (30 nM; AmBic). Disulphide bonds were reduced by adding Tris (2-carboxyethyl) phosphine (TCEP; 50mM), followed by an incubation at 60°C for 1h. Subsequently, proteins were alkylated by adding iodoacetamide (60mM) and incubated at RT for 30 min in the dark. Lastly, proteins were digested by adding trypsin and samples were incubated overnight at 37°C.

Samples were cleaned on a UltraMicroSpin (The Nest Group, Massachusetts, USA) column according to the manufacturers protocol. Peptides were then dried in a miVac DNA concentrator for 1 h. Tryptic peptides were subsequently resuspended in ammonium formate (25 mM) in 85% *v/v* acetonitrile and added to the column, which was centrifuged for 1 min at 110xg. After discarding the supernatant, columns were washed by adding ammonium format in 85% *v/v* acetonitrile. Samples were eluted by placing the columns onto new 2 mL microcentrifuge tubes, and by adding 30 µL of 15 mM formic acid, followed by a 1 min centrifugation at 110 xg. The elution was repeated one more time to maximize sample yield.

Samples were analyzed using SCIEX (Framingham, Boston, MA, USA) TripleTOF 6600 instrumentation coupled on-line to an Eksigent LC system with a nanoLC 400 autosampler and nanoLC 425 pump module, according to the standard operating procedures of the Stoller Biomarker Discovery Centre (Manchester, U.K.). A YMC-Triart C18 trap column and a YMC-Triart C18 analytical column (YMC, Japan) were used to resolve peptides. Peptides were eluted using a gradient from a 98% *v/v* water, 2% *v/v* acetonitrile and 0.1% *v/v* formic acid buffer to an 80% (v/v)

acetonitrile, 20% (v/v) water, 0.1% (v/v) formic acid buffer over 120 minutes at 0.3uL/min.

Nuclear and cytosolic samples from control and RUNX1::RUNX1T1 cells were analyzed using the data-independent acquisition (DIA) method. Raw spectral data files were analyzed using openSWATH (version 2.0.0) using the Pan Human Library [6] followed by scoring and filtering using pyProphet (version 0.18.3). Resultant output files were combined using the MSproteomicstools feature alignment script. Further filtering and protein abundance summarization and normalization were performed in R (version 3.4.1) using the packages SWATH2stats and MSstats from Bioconductor (release 3.5).

Statistical and data analysis

Statistical analysis was performed using a paired sample t-test, assuming equal variances, a one-way ANOVA with Bonferroni's multiple test correction, or a Mann-Whitney test, unless otherwise stated. Values of $p<0.05$ were considered statistically significant using at least three independent biological replicates. All statistical analyses were conducted using GraphPad Prism (ver. 8) (GraphPad Software, California, USA).

Partek Genomics Suite® (v7, St. Louis, USA) was used to perform statistical analysis on the protein expression dataset. An ANOVA was used to determine statistically ($p<0.05$) significant protein changes between RUNX1::RUNX1T1 expressing and control cells. Mean values of protein expression were used for calculation of fold-change (FC). The Metacore™ Pathway Analysis tool (Clarivate) was used to perform Enrichment analyses of the dataset of dysregulated proteins. The Metacore™ Interactome tool was used to identify proteins overrepresented within the given datasets.

Gene expression data was obtained from GSE42519 [7], GSE13159 [8], GSE15434 [9], GSE61804 [10], GSE14468 [11], E-MEXP-583 [1] and TCGA [2] datasets.

References

1. Tonks A, Pearn L, Musson M, et al. Transcriptional dysregulation mediated by RUNX1-RUNX1T1 in normal human progenitor cells and in acute myeloid leukaemia. *Leukemia*. Dec 2007;21(12):2495–505. doi:10.1038/sj.leu.2404961

2. Ley TJ, Miller C, Ding L, et al. Genomic and epigenomic landscapes of adult de novo acute myeloid leukemia. *N Engl J Med*. 05 2013;368(22):2059–74. doi:10.1056/NEJMoa1301689

3. Tonks A, Pearn L, Tonks AJ, et al. The AML1-ETO fusion gene promotes extensive self-renewal of human primary erythroid cells. *Blood*. Jan 2003;101(2):624–32. doi:10.1182/blood-2002-06-1732

4. Tonks A, Tonks AJ, Pearn L, et al. Expression of AML1-ETO in human myelomonocytic cells selectively inhibits granulocytic differentiation and promotes their self-renewal. *Leukemia*. Jul 2004;18(7):1238–45. doi:10.1038/sj.leu.2403396

5. Menezes AC, Jones R, Shrestha A, et al. Increased expression of RUNX3 inhibits normal human myeloid development. *Leukemia*. Jul 2022;36(7):1769–1780. doi:10.1038/s41375-022-01577-2

6. Rosenberger G, Koh CC, Guo T, et al. A repository of assays to quantify 10,000 human proteins by SWATH-MS. *Sci Data*. 2014;1:140031. doi:10.1038/sdata.2014.31

7. Rapin N, Bagger FO, Jendholm J, et al. Comparing cancer vs normal gene expression profiles identifies new disease entities and common transcriptional programs in AML patients. *Blood*. Feb 2014;123(6):894–904. doi:10.1182/blood-2013-02-485771

8. Haferlach T, Kohlmann A, Wieczorek L, et al. Clinical utility of microarray-based gene expression profiling in the diagnosis and subclassification of leukemia: report from the International Microarray Innovations in Leukemia Study Group. *J Clin Oncol*. May 20 2010;28(15):2529–37.

9. Klein HU, Ruckert C, Kohlmann A, et al. Quantitative comparison of microarray experiments with published leukemia related gene expression signatures. *BMC Bioinformatics*. Dec 2009;10:422. doi:10.1186/1471-2105-10-422

10. Metzelder SK, Michel C, von Bonin M, et al. NFATc1 as a therapeutic target in FLT3-ITD-positive AML. *Leukemia*. Jul 2015;29(7):1470–7. doi:10.1038/leu.2015.95

11. Wouters BJ, Löwenberg B, Erpelinck-Verschueren CA, van Putten WL, Valk PJ, Delwel R. Double CEBPA mutations, but not single CEBPA mutations, define a subgroup of

acute myeloid leukemia with a distinctive gene expression profile that is uniquely associated with a favorable outcome. *Blood*. Mar 2009;113(13):3088–91. doi:10.1182/blood-2008-09-179895
